# Intravenous Bacille Calmette–Guérin vaccination protects simian immunodeficiency virus-infected macaques from tuberculosis

**DOI:** 10.1038/s41564-023-01503-x

**Published:** 2023-10-09

**Authors:** Erica C. Larson, Amy L. Ellis-Connell, Mark A. Rodgers, Abigail K. Gubernat, Janelle L. Gleim, Ryan V. Moriarty, Alexis J. Balgeman, Cassaundra L. Ameel, Solomon Jauro, Jaime A. Tomko, Kara B. Kracinovsky, Pauline Maiello, H. Jake Borish, Alexander G. White, Edwin Klein, Allison N. Bucsan, Patricia A. Darrah, Robert A. Seder, Mario Roederer, Philana Ling Lin, JoAnne L. Flynn, Shelby L. O’Connor, Charles A. Scanga

**Affiliations:** 1 Department of Microbiology and Molecular Genetics, School of Medicine, University of Pittsburgh, Pittsburgh, PA USA; 2 Center for Vaccine Research, School of Medicine, University of Pittsburgh, Pittsburgh, PA USA; 3 Department of Pathology and Laboratory Medicine, University of Wisconsin, Madison, WI USA; 4 Division of Laboratory Animal Resources, School of Medicine, University of Pittsburgh, Pittsburgh, PA USA; 5 Vaccine Research Center, National Institute of Allergy and Infectious Diseases, National Institutes of Health, Bethesda, MD USA; 6 Department of Pediatrics, Children’s Hospital of Pittsburgh, School of Medicine, University of Pittsburgh, Pittsburgh, PA USA; 7 Wisconsin National Primate Research Center, University of Wisconsin, Madison, WI USA

**Keywords:** Tuberculosis, HIV infections, Vaccines

## Abstract

Tuberculosis, caused by *Mycobacterium tuberculosis* (Mtb), is the most common cause of death in people living with human immunodeficiency virus (HIV). Intra-dermal Bacille Calmette–Guérin (BCG) delivery is the only licensed vaccine against tuberculosis; however, it offers little protection from pulmonary tuberculosis in adults and is contraindicated in people living with HIV. Intravenous BCG confers protection against Mtb infection in rhesus macaques; we hypothesized that it might prevent tuberculosis in simian immunodeficiency virus (SIV)-infected macaques, a model for HIV infection. Here intravenous BCG-elicited robust airway T cell influx and elevated plasma and airway antibody titres in both SIV-infected and naive animals. Following Mtb challenge, all 7 vaccinated SIV-naive and 9 out of 12 vaccinated SIV-infected animals were protected, without any culturable bacteria detected from tissues. Peripheral blood mononuclear cell responses post-challenge indicated early clearance of Mtb in vaccinated animals, regardless of SIV infection. These data support that intravenous BCG is immunogenic and efficacious in SIV-infected animals.

## Abstract (web summary)

Intravenous administration of the BCG vaccine protects non-human primates with pre-existing SIV infection from tuberculosis.

## Main

*Mycobacterium tuberculosis* (Mtb) infection results in over ten million cases of active tuberculosis (TB) worldwide every year^1^. People living with human immunodeficiency virus (PLWHIV) are very susceptible to Mtb infection and TB disease^2^. TB rapidly progresses and accounts for one in every three deaths among PLWHIV^3^. Although TB is treatable, complications in PLWHIV such as drug–drug interactions with anti-retroviral therapy (ART), issues with compliance due to adverse drug effects, access to healthcare and development of TB–immune reconstitution inflammatory syndrome remain a barrier to successful TB treatment in this vulnerable population. Vaccines are the best public health approach to prevent infectious diseases such as TB, especially in resource-poor settings. However, safety, immunogenicity and efficacy are a concern in PLWHIV due to immunosuppression from chronic HIV infection.

Bacille Calmette–Guérin (BCG) is the only vaccine currently licensed for TB prevention. BCG is a live attenuated *Mycobacterium* *bovis* strain delivered intra-dermally at birth. It confers excellent protection against disseminated TB in children (for example, meningeal TB and extrapulmonary TB)^4^; however, BCG provides little protection against transmissible pulmonary TB in adolescents and adults, suggesting waning efficacy^5^. The implementation of BCG as a vaccine has remained largely unchanged since its discovery over 100 years ago, and it continues to be one of the most widely used vaccines in the world. There is a critical need to improve vaccine efficacy against pulmonary TB in adolescents and adults. However, BCG is contraindicated in people who are immunosuppressed, such as PLWHIV^6^. The exception to this contraindication is HIV-infected individuals who are receiving ART, are clinically well and have a CD4 T cell count of >200 cells mm^−3^ (ref. ^7^). It is imperative that vaccines are developed that are not only safe and effective for immunocompetent individuals, but also protect susceptible populations^4^.

Several groups have investigated different ways to boost protection conferred by BCG, such as subunit vaccines, co-administration with adenoviral vectors encoding Mtb proteins, and use of recombinant strains of BCG^8–11^. However, these efforts were unsuccessful at achieving robust or sterilizing immunity. Studies in mice^12^ and non-human primates (NHP)^13^ showed that concurrent infection with Mtb confers protection from a secondary Mtb challenge, suggesting that high-level protection against Mtb is possible. Recently, substantial protection against TB has been achieved in macaque models of TB using several approaches including cytomegalovirus-vectored delivery of TB antigens^14^ and mucosal delivery of BCG^15^. Recent work by our group and others demonstrated that intravenous (IV) BCG immunization confers robust protection against TB in rhesus macaques^16–19^. Notably, IV delivery of BCG protected nine out of ten macaques from TB disease (<100 Mtb colony-forming units (CFU)), with six out of ten animals exhibiting sterilizing immunity (undetectable CFU)^16^. IV BCG-elicited protection was associated with high levels of mycobacteria-specific T cells in airways, tissue-resident memory T cells^16^ and robust immunoglobulin (Ig) M responses^20^.

While the above studies in immunocompetent macaques generated renewed enthusiasm for TB vaccines, these vaccines have not been evaluated in the setting of immune suppression, such as simian immunodeficiency virus (SIV) infection. Modelling PLWHIV merits special consideration especially for live attenuated vaccines such as BCG. It remains unknown whether IV delivery of BCG is safe and immunogenic in SIV-infected NHP, and whether the robust protection conferred by IV BCG in healthy animals would be recapitulated in immunocompromised macaques.

In this Article, we extended the studies of Darrah et al.^16^ to determine whether IV BCG immunization is safe, immunogenic and protective in our established model of SIV/Mtb co-infection in Mauritian cynomolgus macaques (MCM). MCM have similar high susceptibility to Mtb infection and disease as rhesus macaques^21^ and SIV infection exacerbates TB progression in this species^22^. We show that MCM chronically infected with SIV can be vaccinated with high-dose IV BCG without adverse effects. Furthermore, IV BCG elicits strong immune responses in both SIV-infected and SIV-naive macaques. MCM responses were consistent with those reported in rhesus macaques^16^, including the induction of T cells in the airways as well as robust antibody responses in blood and airways. Most strikingly, IV BCG conferred robust protection against Mtb challenge irrespective of SIV infection status–providing sterilizing immunity in all 7 SIV-naive animals and 9 out of 12 SIV-infected animals.

## Results

### IV BCG is safe in SIV-infected animals

**Extended Data Fig. 1:**
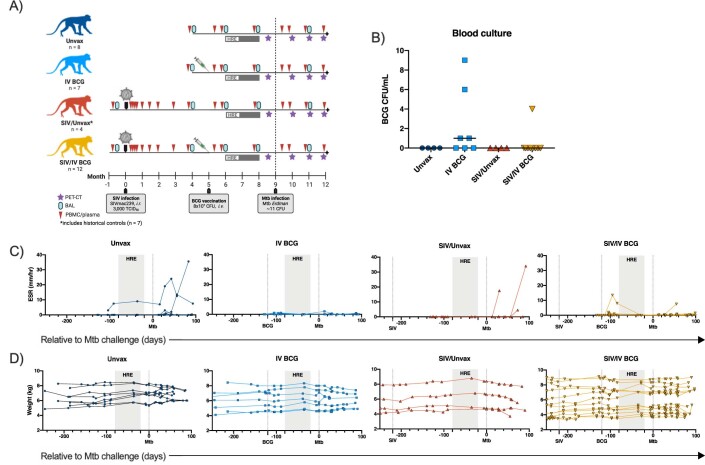
Study outline and clinical parameters. **a)** Timeline of NHP groups including SIV infection, BCG vaccination, HRE therapy, and Mtb challenge. Sampling schedule of blood draws, BAL, and PET-CT image is also included. Created with BioRender.com. **b)** BCG CFU/mL of blood cultures collected 2 wks after vaccination. Horizontal bars indicate group medians and symbols indicate individual animals (Unvax, n = 4; IV BCG, n = 7; SIV/Unvax, n = 4; SIV/IV BCG, n = 12). **c)** ESR (mm/hr) of individual animals in each group relative to Mtb challenge. **d)** Individual animal weights (kg) of each group relative to Mtb challenge. C & D) Symbols indicate individual animals (Unvax, n = 8; IV BCG, n = 7; SIV/Unvax, n = 4; SIV/IV BCG, n = 12). Source data

We sought to determine whether IV BCG was safe, immunogenic and efficacious in animals with a pre-existing, chronic SIV infection using our established model of SIV/Mtb co-infection in MCM^22,23^. This study included four groups of animals: SIV-naive, unvaccinated (Unvax) (*N* = 8); SIV-naive, vaccinated (IV BCG) (*N* = 7); SIV+, unvaccinated (SIV/Unvax) (*N* = 4; historical controls included for TB outcome only, *N* = 7); and SIV+, vaccinated (SIV/IV BCG) (*N* = 12) (Extended Data Fig. 1a). For the SIV+ groups, animals were intrarectally infected with SIVmac239 (3,000 50% tissue culture infectious dose). Five months later, some animals were vaccinated intravenously with BCG at 8 × 10^7^ CFU. Due to concerns over potential disseminated BCG disease in immunocompromised animals, animals were treated with an 8-week regimen of isoniazid/rifampin/ethambutol (HRE) 3–4 weeks after IV BCG vaccination. Unvaccinated animals also received HRE to minimize any confounding effects due to antibiotic therapy. Four weeks after antibiotic cessation (4 months post-BCG in vaccinated animals), all animals were challenged with low-dose Mtb Erdman (~11 CFU) via bronchoscope, followed for 3 months and then necropsied.

To assess the safety of IV BCG, blood cultures were performed 2 weeks after vaccination, and erythrocyte sedimentation rate (ESR), an indicator of inflammation, and weight were measured over the course of vaccination. A total of 4 out of the 7 SIV-naive, vaccinated animals, and 1 out of the 12 SIV+ vaccinated animals, had culturable BCG in their blood 2 weeks after vaccination (Extended Data Fig. 1b). One animal in the SIV+ vaccinated group had a transiently increased ESR ~1 month post-vaccination (Extended Data Fig. 1c). There were no notable changes in clinical status, ESR or weight in the remainder of the animals following vaccination (Extended Data Fig. 1c,d). Thus, based on these clinical measures, IV BCG appears to be safe in SIV+ macaques, at least when treated with HRE within 3–4 weeks after administration.

**Extended Data Fig. 2:**
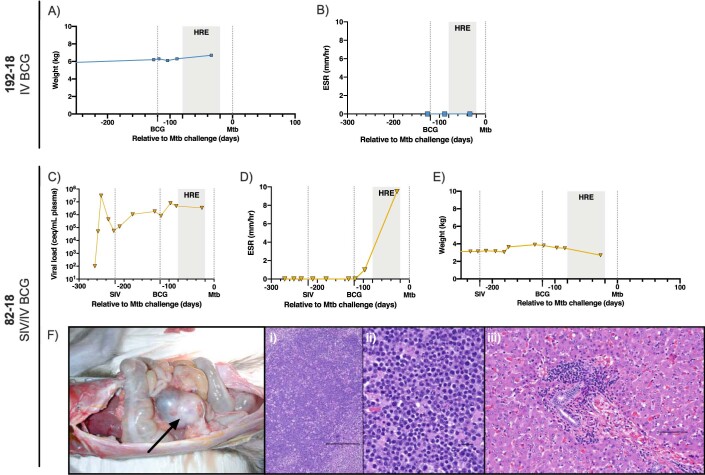
Clinical parameters of 192-18 and 82-18. **a)** Weight of 192-18 over time relative to Mtb challenge. **b)** ESR of 192-18 over time. **c)** Weight of 82-18 over time relative to Mtb challenge. **d)** ESR of 82-18 over time. **e)** Plasma viral load over course of study. **f)** Histopathological signs of lymphoma in 82-18. Photomicrographs were representative of 30 slides examined per animal. Left panel: image of ileocecocolic mass (black arrow). Middle panel (i & ii): 10X (left; scale bar = 100 μm) and 40X (right; scale bar = 10 μm) H&E-stained section of ileocecocolic mass indicating large non-cleaved lymphoid population. Right panel (iii): H&E-stained section of liver (20X), peri-portal lymphoid infiltration (scale bar = 50 μm). Source data

It is worth noting that two IV BCG-vaccinated animals did not reach Mtb challenge, probably due to reasons unrelated to BCG. The first animal, 192-18, was SIV-naive and died 12 weeks after vaccination due to a presumed fatal arrhythmia during bronchoalveolar lavage (BAL) with no history of weight loss or elevated ESR (Extended Data Fig. 2a,b). The second, 82-18, was SIV-infected and had persistently high plasma viraemia (Extended Data Fig. 2c). Following IV BCG, it exhibited weight loss and elevated ESR (Extended Data Fig. 2d,e). Rapid clinical deterioration ~12 weeks after vaccination led to euthanasia and necropsy where a large abdominal lymphoma was identified (Extended Data Fig. 2f). Neither animal had detectable BCG by blood culture 2 weeks after vaccination, and 82-18 showed no histopathological signs of granulomatous disease. Data from these animals were excluded from further analysis.

### IV BCG triggers a transient burst of SIV replication

**Fig. 1:**
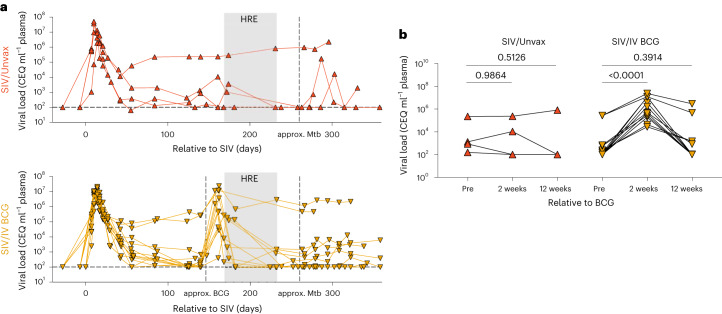
Plasma viraemia of SIV+ unvaccinated and vaccinated animals. Plasma viral copy equivalents were determined by qRT–PCR. Each point indicates an individual animal (SIV/Unvax, *n* = 4; SIV/IV BCG, *n* = 12). **a**, Plasma viral load (in copy equivalents (CEQ) ml^−1^) of SIV+ unvaccinated (top, red up-pointing triangles) and SIV+ vaccinated (bottom, gold down-pointing triangles) animals over the course of the study. Horizontal dashed line represents the limit of detection. **b**, Plasma viral load of each group before (pre), 2 weeks and 12 weeks, relative to BCG vaccination. Time-matched plasma from SIV+ unvaccinated animals served as a control. Repeated measure one-way ANOVAs were performed on each group (SIV/Unvax, *P* = 0.5759; SIV/IV BCG, *P* < 0.0001). Multiple comparisons relative to ‘pre-BCG’ were performed using Dunnett’s multiple comparisons tests. Adjusted *P* values for multiple comparisons are shown. Source data

Plasma viraemia was assessed serially in vaccinated and unvaccinated SIV+ animals. All animals in this study had at least one copy of the major histocompatibility complex (MHC) haplotype M1. This ensures all animals shared at least one copy of the MHC alleles of the M1 haplotype, so that all animals should mount a population of mycobacterial-specific T cells targeting the same epitopes^24^. As previously reported^25^, M1+ animals display either spontaneously controlled viraemia (<10^4^ copies ml^−1^) or high levels of viraemia (>10^5^ copies ml^−1^). As expected, plasma viraemia peaked 1–2 weeks post SIV infection and by 7–8 weeks post-infection, animals showed a range of viral set points. There was a spike in plasma viraemia ~2 weeks following BCG vaccination (Fig. 1a). This increase in plasma viraemia was significant in SIV+ vaccinated animals compared with time-matched SIV+ unvaccinated animals (Fig. 1b). Plasma viraemia in SIV+ vaccinated animals returned to their pre-vaccination levels during HRE treatment, before Mtb challenge (Fig. 1b). Following Mtb challenge, we did not observe a consistent effect on plasma viraemia as reported previously^22^. These data indicate that IV BCG was a potent stimulator of viral replication; however, this effect was transient.

### IV BCG induces mycobacterial-specific T cell airway influx

**Fig. 2:**
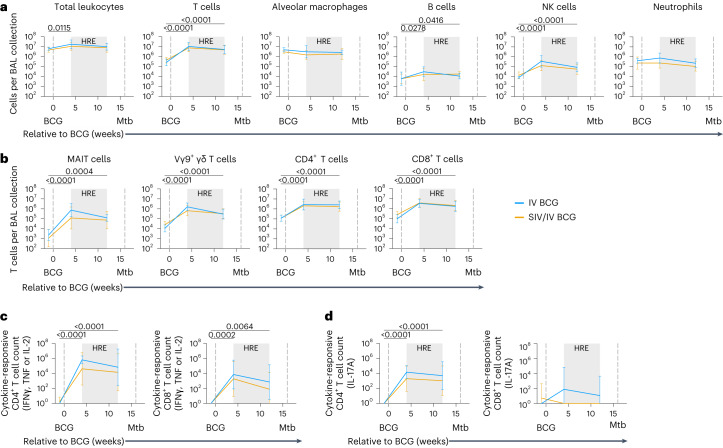
Leukocyte and T cell subsets and T cell response in BAL after BCG vaccination. **a**, Number of leukocytes per BAL collection. **b**, Number of T cell subset cells per BAL collection. **c**, Number of cytokine-responsive (IFNγ, TNF and IL-2) CD4^+^ and CD8^+^ T cells in BAL after 14 h stimulation with PPD. **d**, Number of IL-17A-responsive CD4^+^ and CD8^+^ T cells in BAL after 14 h stimulation with PPD. Data are mean and s.d. of SIV-naive (light blue) and SIV+ (gold) vaccinated animals (**a**–**d**). SIV-naive vaccinated animals: pre-BCG (*n* = 5), 4 weeks post-BCG (*n* = 6) and 12 weeks post-BCG (*n* = 6). SIV+ vaccinated animals: pre-BCG (*n* = 10), 4 weeks post-BCG (*n* = 11) and 12 weeks post-BCG (*n* = 8). Individual animal data are shown in Supplementary Data 5. Mixed effects models with subject as a random variable were used to assess mean differences among timepoints and vaccine groups. Timepoints were compared with ‘pre-BCG’ control using Dunnett’s multiple comparison tests. No significant differences between vaccination groups were found. Significant *P* values (*P* < 0.05) across time are shown above each graph. Fixed effect test results and Dunnett’s multiple comparisons are included in Supplementary Table 2a. All statistical tests were two-sided. Source data

Since the primary site of Mtb infection is the lung, we assessed whether IV BCG vaccination led to enhanced immune responses in airways of SIV+ and SIV-naive MCM as previously described in SIV-naive rhesus macaques^16^. Flow cytometry on serial BAL showed a striking influx of T cells into the airways 4 weeks after IV BCG in both SIV-naive and SIV+ animals (Fig. 2a). This high number of T cells was sustained 12 weeks after vaccination (1 month before Mtb challenge), regardless of SIV status. There was also a significant influx of B cells and natural killer (NK) cells in both vaccinated groups (Fig. 2a). Mucosal-associated invariant T (MAIT) cells, Vγ9^+^ γδ T cells, CD4^+^ T cells and CD8^+^ T cells significantly increased in number following vaccination and were maintained 12 weeks after vaccination, just before Mtb challenge (Fig. 2b).

Next, we assessed whether the T cells present in the airways of vaccinated animals were able to produce cytokines associated with Mtb control (interferon gamma (IFNγ), tumour necrosis factor (TNF), interleukin-2 (IL-2)^26–28^ in response to mycobacterial-specific antigens (purified protein derivative (PPD)). There was a significant increase in the number of cytokine-producing CD4^+^ T cells, reaching 10^4^–10^6^ cells, in the airways of SIV-naive and SIV+ vaccinated animals at 4 and 12 weeks after vaccination (Fig. 2c). Cytokine-producing CD8^+^ T cells were significantly elevated, albeit lower than the CD4^+^ T cells, following vaccination in both BCG-vaccinated groups (Fig. 2c). There were increased numbers of mycobacterial-responsive CD4^+^ T cells producing IL-17A, a cytokine associated with mucosal immunity^29^, in the airways of both SIV-naive and SIV+ macaques following vaccination (Fig. 2d). Mycobacterial-responsive CD8^+^ T cells producing IL-17A was very low in both groups (Fig. 2d). Together, these data indicate that IV BCG vaccination induces a rapid and sustained influx of mycobacterial-specific T cells into the airways, regardless of SIV status.

### IV BCG induces mycobacterial-specific responses in blood

**Fig. 3:**
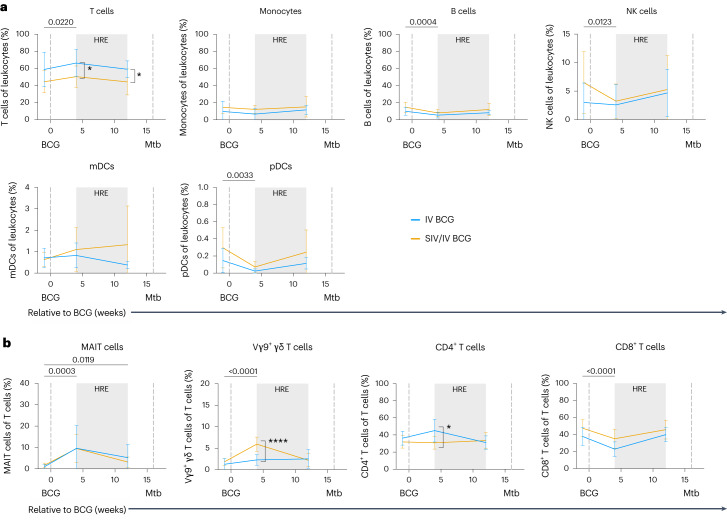
Frequencies of leukocyte and T cell subsets in PBMC after vaccination. **a**, Frequencies of leukocyte subsets relative to BCG. mDCs, myeloid dendritic cells; pDCs, plasmacytoid dendritic cells. **b**, Frequencies of T cells and T cell subsets (MAIT, Vγ9^+^, CD4^+^ and CD8^+^ T cells) relative to BCG. Data are mean and s.d. of SIV-naive (IV BCG, light blue) and SIV+ (SIV/IV BCG, gold) vaccinated animals. SIV-naive vaccinated animals: pre-BCG (*n* = 7), 4 weeks post-BCG (*n* = 7) and 12 weeks post-BCG (*n* = 7). SIV+ vaccinated animals: pre-BCG (*n* = 12), 4 weeks post-BCG (*n* = 12) and 12 weeks post-BCG (*n* = 11). Individual animal data are shown in Supplementary Data 6. Mixed effects models with subject as a random variable were used to assess mean differences among timepoints and vaccine groups. Timepoints were compared to ‘pre-BCG’ control using Dunnett’s multiple comparison tests. Significant *P* values (*P* < 0.05) across time are shown above each graph. Significant differences determined by Mann–Whitney tests between vaccination groups at given timepoints are indicated by brackets and significant *P* values (not adjusted for multiple comparison) indicated: *P < 0.05 and ****P < 0.0001. Fixed effect test results, Dunnett’s multiple comparisons and Mann–Whitney test results are reported in Supplementary Table 2b. All statistical tests were two-sided. Source data

We assessed the circulating immune cell landscape and T cell responses to mycobacterial and Mtb-specific stimuli in peripheral blood mononuclear cells (PBMC). There was a modest but significant increase in the frequency of T cells, along with a small decline in B cell, NK cell and plasmacytoid dendritic cell populations, following BCG vaccination at 4 weeks (Fig. 3a). The frequency of MAIT cells significantly increased 4 weeks after vaccination and was maintained 12 weeks after vaccination, regardless of SIV status (Fig. 3b). Vγ9^+^ γδ T cells significantly increased 4 weeks after BCG vaccination in both vaccinated groups (Fig. 3b). However, SIV+ animals had significantly higher frequencies of Vγ9^+^ γδ T cells compared with SIV-naive animals (Fig. 3b). Vγ9^+^ γδ T cell frequencies returned to pre-infection levels by 12 weeks post-BCG. The frequency of CD4^+^ T cells did not change significantly over vaccination (Fig. 3b), although CD4^+^ T cell frequencies were significantly lower in SIV+ compared with SIV-naive animals 4 weeks after IV BCG (Fig. 3b), most probably due to the transient spike in SIV replication^30^. CD8^+^ T cells, on the other hand, significantly declined in both vaccinated groups 4 weeks after BCG vaccination (Fig. 3b). Frequencies of both CD4^+^ and CD8^+^ T cells returned to near pre-vaccination baseline levels by week 12 after IV BCG.

**Extended Data Fig. 3:**
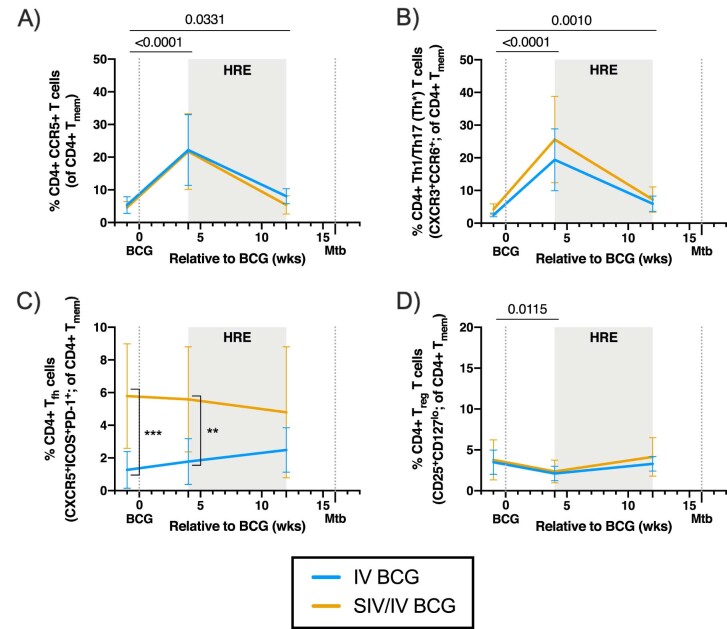
CD4 + T cell phenotype in PBMC after vaccination. **a)** Frequency of CD4 + CCR5 + T cells in PBMC relative to BCG vaccination. **b)** Frequency of CD4 + Th1/Th17 (Th*) T cells (CXCR3^+^CCR6^+^) in PBMC relative to BCG vaccination. **c)** Frequency of CD4+ T_fh_ T cells (CXCR5^+^ICOS^+^PD-1^+^) in PBMC relative to BCG vaccination. **d)** Frequency of CD4+ T_reg_ T cells (CD25^+^CD127^lo^) in PBMC relative to BCG vaccination. **a-d)** Lines indicate mean and error bars indicate SD of SIV-naïve (light blue) and SIV+ (gold) vaccinated animals. SIV-naïve vaccinated animals: pre-BCG (n = 7), 4 wks post BCG (n = 7), and 12 wks post BCG (n = 7). SIV+ vaccinated animals: pre-BCG (n = 12), 4 wks post BCG (n = 12), and 12 wks post BCG (n = 11). Individual animal data are shown in Supplementary Data 8. Linear mixed effects models with subject as a random variable were used. Fixed effect tests were used to assess mean differences among time points and vaccine groups. Time points were compared to ‘pre-BCG’ control using Dunnett’s multiple comparison tests. Significant p-values (p < 0.05) across time are shown above each graph. Significant differences determined by Mann-Whitney tests between vaccination groups at given time points are indicated by brackets and significant p-values indicated: **p < 0.01 and ***p < 0.001. Fixed effect test results, Dunnett’s multiple comparisons, and Mann-Whitney test results are reported in Supplementary Table 2C. All statistical tests were two-sided. Source data

CCR5^+^ and CXCR3^+^CCR6^+^ CD4^+^ T cell frequencies peaked in PBMC 4 weeks after vaccination and remained elevated compared with pre-BCG regardless of SIV status (Extended Data Fig. 3a,b). CCR5 is expressed on effector T cells and is a co-receptor the facilitates HIV/SIV infection^31,32^, whereas T cells expressing both CXCR3 and CCR6 are associated with T helper 1 (T_H_1)/T_H_17 (T_H_1*) responses^33,34^. In addition, the frequency of circulating CD4^+^ T follicular helper (T_FH_) cells in SIV+ vaccinated animals were elevated across all timepoints, including pre-BCG, compared with SIV-naive animals, probably due to chronic SIV (Extended Data Fig. 3c). There was a small but significant reduction in CD4^+^ T regulatory (T_reg_) cells in both vaccinated groups 4 weeks after IV BCG (Extended Data Fig. 3d).

**Fig. 4:**
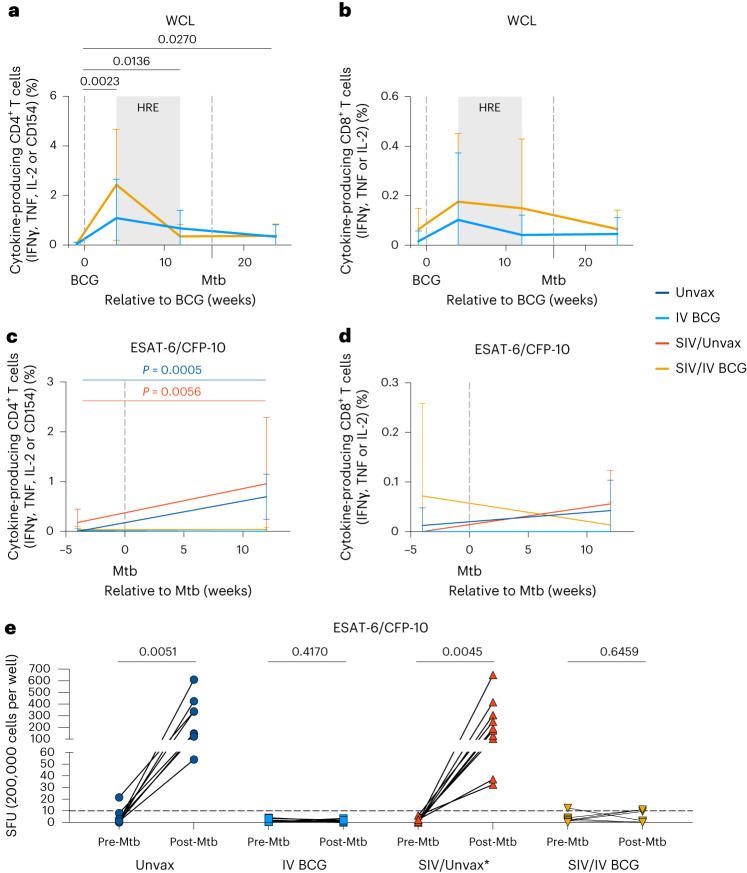
T cell response in PBMC after BCG vaccination and Mtb challenge. **a**,**b**, Frequency of cytokine-responsive CD4^+^ (**a**) and CD8^+^ (**b**) T cells in PBMC after 14 h stimulation with H37Rv whole cell lysate relative to BCG vaccination. Mixed effects models with subject as a random variable were used to assess mean differences among timepoints and vaccine groups. Timepoints were compared with ‘pre-BCG’ control using Dunnett’s multiple comparison tests. Significant *P* values (*P* < 0.05) across time are shown above each graph. Fixed effect test results and Dunnett’s multiple comparisons are reported in Supplementary Table 2d. **c**,**d**, Frequency of cytokine-responsive CD4^+^ (**c**) and CD8^+^ (**d**) T cells in PBMC after 14 h stimulation with ESAT-6/CFP-10. Data shown in weeks relative to Mtb challenge. Linear mixed effects models were used to determine significant mean differences between time and animal group. Šidák’s multiple comparisons test *P* values are reported in Supplementary Table 2d. Significant *P* values (*P* < 0.05) across time for each treatment group are shown above each graph. In **a**–**d**, data are mean and s.d. of Unvax (dark blue), IV BCG (light blue), SIV/Unvax (red) and SIV/IV BCG (gold) animals. Unvaccinated animals (Unvax): pre-Mtb (*n* = 8) and 12 weeks post-Mtb (*n* = 8). SIV-naive vaccinated animals (IV BCG): pre-BCG (*n* = 7), 4 weeks post-BCG (*n* = 7), 12 weeks post-BCG/pre-Mtb (*n* = 7) and 12 weeks post-Mtb (*n* = 7). SIV+ unvaccinated (SIV/Unvax): pre-Mtb (*n* = 4) and 12 weeks post-Mtb (*n* = 4). SIV+ vaccinated animals (SIV/IV BCG): pre-BCG (*n* = 12), 4 weeks post-BCG (*n* = 12), 12 weeks post-BCG/pre-Mtb (*n* = 11) and 12 weeks post-Mtb (*n* = 12). Individual animal data are shown in Supplementary Data 7. **e**, IFNγ production in PBMC before and after Mtb challenge (12 weeks post-Mtb) by ELISpot. SFU, spot-forming units. Individual symbols and lines indicate individual animals. Unvax, pre-Mtb (*n* = 8) and post-Mtb (*n* = 8); IV BCG, pre-BCG (*n* = 7) and post-Mtb (*n* = 7); SIV/Unvax with historical controls, pre-Mtb (*n* = 8) and post-Mtb (*n* = 8); SIV/IV BCG, pre-BCG (*n* = 10) and post-Mtb (*n* = 9). Paired *t*-tests were used to determine significance within each group. *P* values are shown. All statistical tests were two-sided. Source data

CD4^+^ and CD8^+^ T cells in blood produced cytokines (IFNγ, TNF and IL-2) and increased expression of CD154, a marker associated with antigen-specificity^35^, upon stimulation to mycobacterial antigens (Mtb whole cell lysate (WCL)) at 4 and 12 weeks post-BCG (Fig. 4a, b), with no significant differences between SIV-naive and SIV+ macaques. Cytokine production by CD4^+^ T cells remained elevated in both groups until 12 weeks post-Mtb challenge (Fig. 4a). However, following Mtb challenge, cytokine production in response to peptide pools of the Mtb-specific antigens early secreted antigenic target-6 (ESAT-6) and cyan fluorescent protein-10 (CFP-10) (antigens not present in BCG) by CD4^+^ T cells was only observed in non-vaccinated SIV-naive or SIV+ macaques (Fig. 4c,d). IFNγ ELISpot assays of PBMC collected pre- and post-Mtb challenge also showed that both unvaccinated groups generated an IFNγ response to Mtb-specific ESAT-6 and CFP-10 (Fig. 4e). Notably, PBMC from vaccinated animals, regardless of SIV status, did not generate an IFNγ response to Mtb antigens (Fig. 4e). These data suggest that IV BCG induces rapid and early clearance of Mtb, precluding a measurable T cell response (IFNγ, TNF or IL-2) to infection, as noted in our previously published study on IV BCG in SIV-naive rhesus macaques^16^.

### IV BCG induces mycobacterial-specific antibody responses

**Fig. 5:**
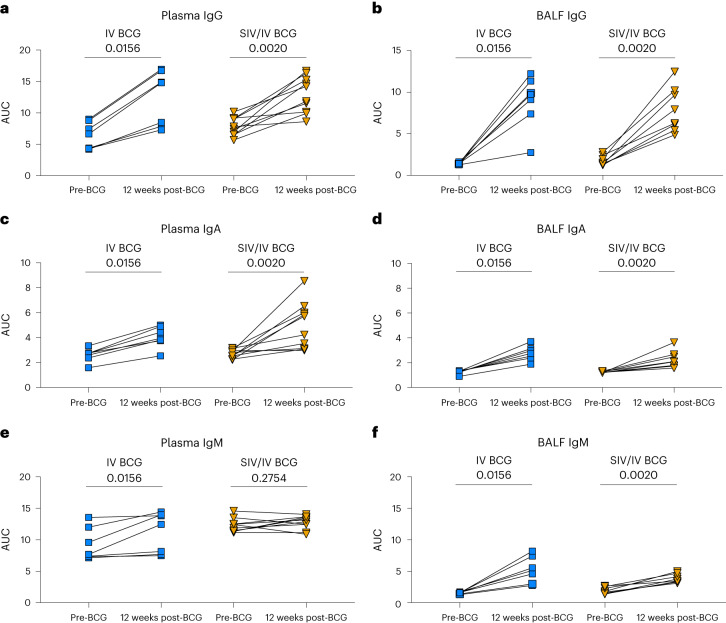
Mycobacterial-specific antibodies in plasma and BALF following BCG vaccination. **a**–**f**, Antibodies specific for H37Rv whole cell lysate were assessed by ELISA for both vaccinated groups: plasma IgG (**a**), BALF IgG (**b**), plasma IgA (**c**), BALF IgA (**d**), plasma IgM (**e**) and BALF IgM (**f**). AUC was determined by calculating the area under the dilution series curve using GraphPad Prism. Individual animals indicated by symbols for IV BCG (light-blue square; *n* = 7) and SIV/IV BCG (gold, down-pointing triangle; *n* = 12). Wilcoxon paired signed rank tests were performed to determine significance. *P* values are shown. Source data

Recent studies have shown that humoral immunity may play role in the control of Mtb infection and is associated with protection by IV BCG^20,36–38^. Humoral responses to mycobacterial antigens (Mtb WCL) in plasma and BAL fluid (BALF) before and 12 weeks after IV BCG vaccination (4 weeks before Mtb challenge) were assessed by enzyme-linked immunosorbent assay (ELISA). There were significant increases in WCL-reactive IgG and IgA levels in the plasma and BALF of both SIV-naive and SIV+ animals following vaccination (Fig. 5a–d). WCL-reactive IgM in plasma increased after vaccination in SIV-naive but not in SIV+ animals (Fig. 5e). It is possible that we were unable to detect a change in plasma IgM in the SIV+ animals due to the high baseline IgM before IV BCG vaccination, which may be due to the inherent pentameric nature of IgM or presence of rheumatoid factor in plasma that contributes to high background levels^39–41^. Nevertheless, there was a significant increase in BALF IgM levels in both groups after IV BCG vaccination (Fig. 5f). Thus, IV BCG induces mycobacterial-specific humoral immune responses in both SIV-naive and SIV+ animals.

### IV BCG protects SIV+ macaques from TB

**Fig. 6:**
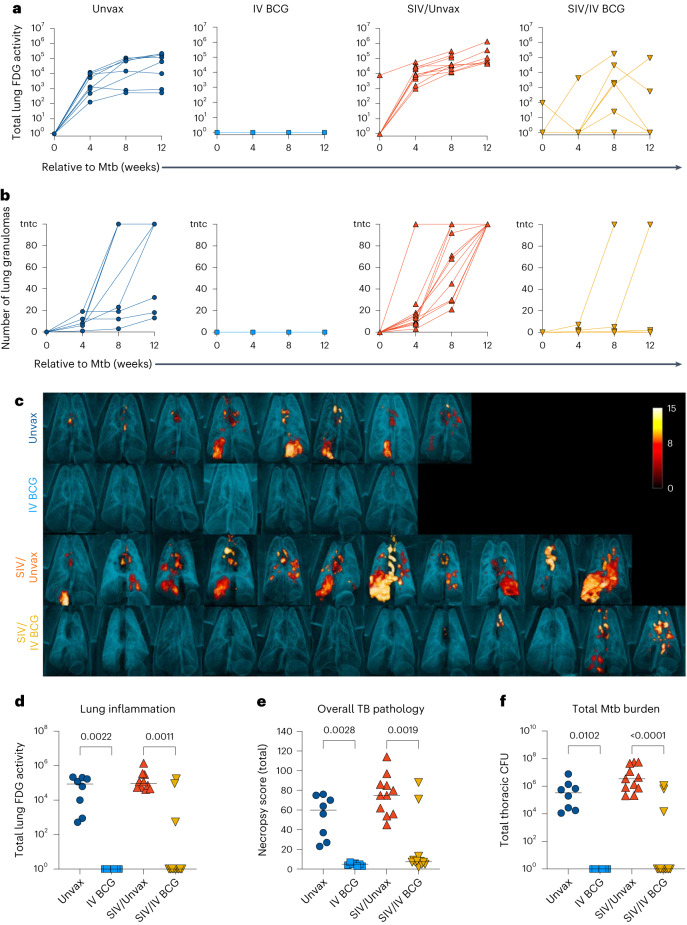
Protection against Mtb challenge in BCG-vaccinated groups. **a**, Total FDG activity (lung inflammation) relative to Mtb challenge, measured by PET/CT imaging. Lines indicate individual animals. **b**, Number of lung granulomas relative to Mtb challenge. Animals with granuloma numbers >100 are indicated as too numerous to count (tntc). At 4 and 8 weeks, granulomas were counted by CT, whereas at 12 weeks, granulomas were counted by gross pathology. **c**, Three-dimensional renderings of PET/CT images of individual animals taken at necropsy. **d**–**f**, Lung inflammation (**d**), overall TB pathology (**e**) and total Mtb burden (thoracic CFU) (**f**) at necropsy. Each point indicates an individual animal and horizontal bars indicate group medians (Unvax, *n* = 8; IV BCG, *n* = 7; SIV/Unvax, *n* = 11; SIV/IV BCG, *n* = 12). Kruskal–Wallis tests were performed with Dunn’s multiple comparisons between SIV-naive, vaccinated and unvaccinated groups (dark-blue circles and light-blue squares, respectively), and SIV+, vaccinated and unvaccinated groups (red, up-pointing triangle and gold, down-pointing triangle, respectively). *P* values are shown. All statistical tests were two-sided. Source data

A total of 4 months after BCG vaccination (1 month after HRE cessation), animals were challenged with low-dose Mtb Erdman via bronchoscope. Inflammation and progression of Mtb infection were serially quantified using ^18^F-fluorodeoxyglucose (FDG) positron emission tomography–computed tomography (PET/CT) imaging. Total lung FDG activity, a surrogate for lung inflammation, revealed substantial lung inflammation in both unvaccinated groups as early as 4 weeks post-Mtb challenge, which remained elevated until necropsy (Fig. 6a). In contrast, SIV-naive vaccinated animals displayed minimal lung inflammation over the 12 weeks of Mtb infection (Fig. 6a). SIV+ vaccinated animals showed variable lung inflammation, especially at 8 weeks post-Mtb challenge (Fig. 6a). We previously showed that dissemination of granulomas, an indicator of TB disease progression, occurred between 4 and 8 weeks after Mtb co-infection in SIV+ animals^22^. We observed a similar effect here (Fig. 6b). IV BCG vaccination of SIV-naive MCM resulted in minimal granuloma formation and no apparent dissemination, recapitulating the results from our prior study in rhesus macaques^16^ (Fig. 6b). Remarkably, most SIV+ vaccinated macaques also had minimal granuloma formation and dissemination, although two animals did show progressive disease by this metric (Fig. 6b). PET/CT imaging before necropsy revealed striking differences in TB disease between the unvaccinated and vaccinated animals (Fig. 6c). Both SIV-naive and SIV+ vaccinated animals had significantly less lung inflammation compared with their respective unvaccinated controls at necropsy, although 3 of the 12 SIV+ vaccinated animals did have increased lung FDG activity (Fig. 6d).

**Extended Data Fig. 4:**
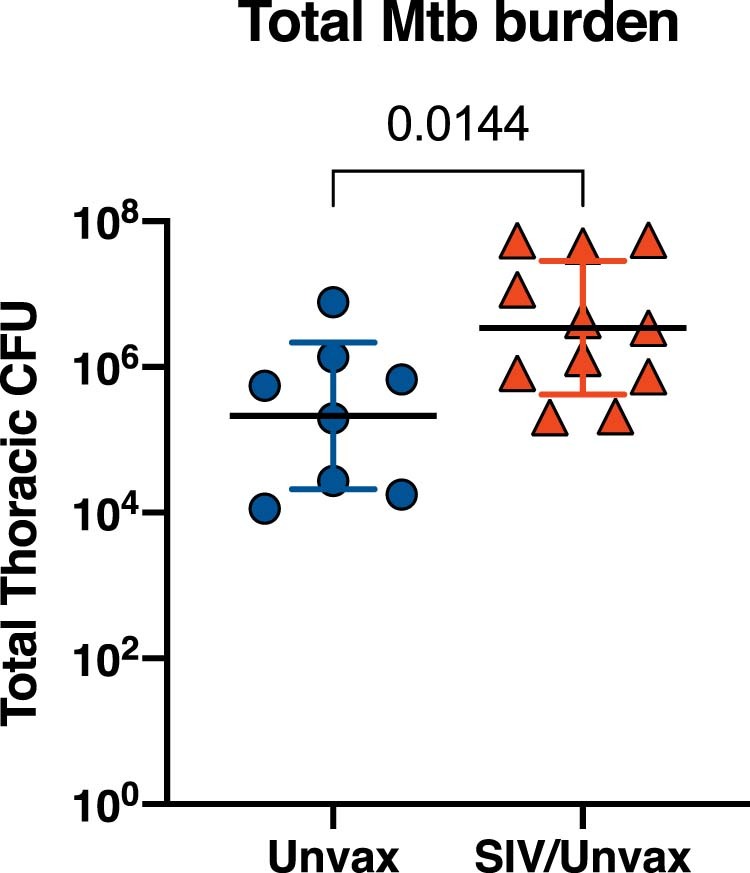
Bacterial burden of SIV-naïve and SIV+ unvaccinated animals. Total thoracic Mtb burden at necropsy. Each point indicates an individual animal, horizontal bars indicate group means, and error bars indicate standard deviation. Unpaired t test (two-sided) was performed between SIV-naïve unvaccinated (Unvax, dark blue circles; n = 8) and SIV+ unvaccinated groups (SIV/Unvax, red, up-pointing triangles; n = 11). P-value is shown. Source data

**Extended Data Fig. 5:**
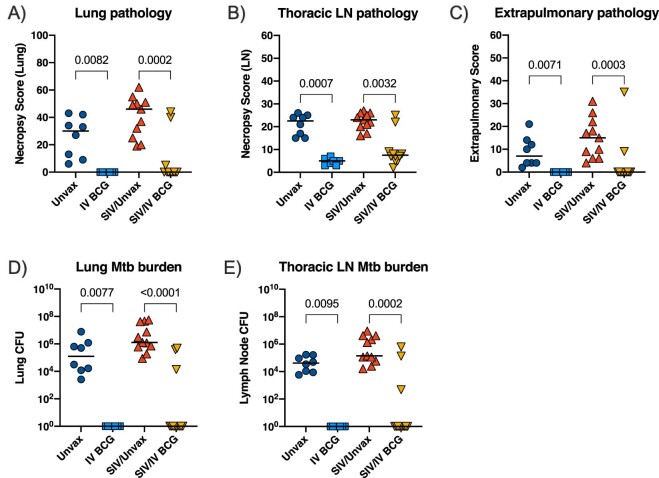
TB outcome by tissue compartment. **a-e)** Lung pathology **(a)**, thoracic lymph node (LN) pathology **(b)**, extrapulmonary pathology **(c)**, lung Mtb burden **(d)**, and thoracic LN Mtb burden **(e)** at necropsy. Each point indicates an individual animal and horizontal bars indicate group medians (Unvax, n = 8; IV BCG, n = 7; SIV/Unvax, n = 11; SIV/IV BCG, n = 12). Kruskal Wallis tests were performed with Dunn’s multiple comparisons between SIV-naïve, vaccinated and unvaccinated groups (dark blue circles and light blue squares, respectively); and SIV+, vaccinated and unvaccinated groups (red, up-pointing triangle and gold, down-pointing triangle, respectively). P-values are shown. All statistical tests were two-sided. Source data

TB pathology was assessed at necropsy using an established scoring system^21^. IV BCG resulted in a significant reduction in lung pathology regardless of SIV, although there were two SIV+ vaccinated animals with high pathology scores (Fig. 6e). Multiple tissue samples were plated at time of necropsy and Mtb bacterial burden (the CFU) was quantified. Tissues were isolated from all 7 SIV-naive vaccinated animals, and 9 out of 12 SIV+ vaccinated animals were completely sterile 12 weeks after Mtb challenge (Fig. 6f). As reported previously^22^, SIV+ unvaccinated animals had significantly higher bacterial burden than SIV-naive unvaccinated animals, indicative of an immune defect due to SIV (Extended Data Fig. 4). IV BCG vaccination resulted in a significant reduction in pathology and Mtb burden across different tissue compartments (lung, thoracic lymph node and extrapulmonary sites), regardless of SIV status (Extended Data Fig. 5a–e). With protection from TB defined as a total thoracic Mtb burden <100 CFU^16^, IV BCG conferred 100% protection in the SIV-naive animals (7 out of 7) and 75% protection in the SIV+ animals (9/12).

**Extended Data Fig. 6:**
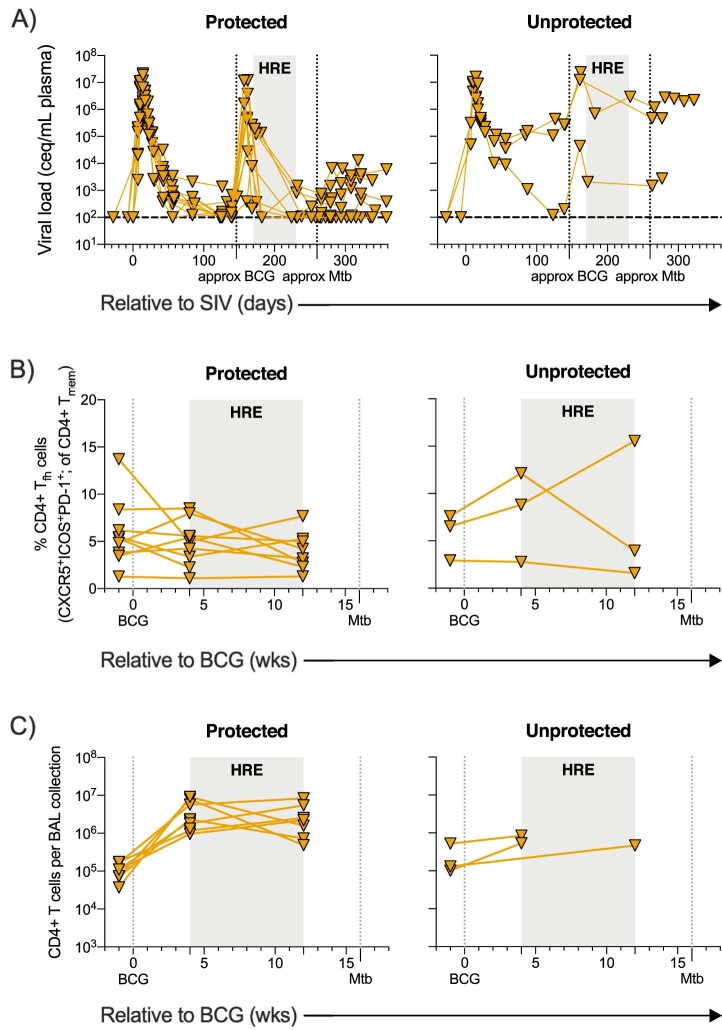
Plasma viral load, PBMC CD4+ T_fh_ T cells, and BAL CD4 + T cell levels stratified by protection across SIV+ vaccinated animals. Individual symbols and lines indicate protected animals (thoracic CFU < 100, n = 9) and unprotected animals (thoracic CFU > 100, n = 3). **a)** Plasma viremia in protected and unprotected SIV+, vaccinated animals over the course of the study. **b)** Frequency of CD4+ T_fh_ T cells (CXCR5^+^ICOS^+^PD-1^+^) in PBMC from protected and unprotected SIV+, vaccinated animals relative to BCG vaccination. **c)** Number of CD4 + T cells per BAL collection in protected and unprotected SIV+, vaccinated animals relative to BCG vaccination. Source data

The three unprotected SIV+ vaccinated animals tended to have higher plasma viraemia levels (Extended Data Fig. 6a). Moreover, these three animals appeared to have elevated levels of circulating CD4^+^ T_FH_ cells and fewer CD4^+^ T cells in the airways during the IV BCG vaccination period (Extended Data Fig. 6b,c). These data suggest that at least two of the unprotected animals showed signs of progressive SIV infection. Progressive SIV impairs immunity, especially through the depletion of CD4^+^ T cells^30,42^. The high viral replication in these animals may have compromised the ability of IV BCG to elicit a successful anti-mycobacterial response, resulting in poor protection against Mtb.

Overall, these data demonstrate that IV BCG vaccination resulted in complete protection against Mtb in SIV-naive animals. Furthermore, we show that high-order protection against Mtb is achievable in SIV+ animals.

## Discussion

Protection against TB in SIV+ macaques has not been previously demonstrated. Here we show that IV BCG can confer sterilizing immunity in 75% of SIV+ macaques. In addition, we recapitulated our previously published robust protection and sterilizing immunity provided by IV BCG in SIV-naive rhesus macaques^16^ in 100% of MCM, a different SIV-naive macaque species. Immune responses to IV BCG were similar in SIV-naive and SIV+ macaques, with a robust and sustained expansion of mycobacterial-specific CD4^+^ and CD8^+^ T cells in the airways, lower but significant increases in γδ T cells and MAIT cells, and enhanced mycobacterial-reactive antibody responses in blood and airways.

HIV/SIV infection is known to impair immune responses in their respective hosts. One concern of HIV/SIV infection is whether infection will interfere with vaccine-elicited immune responses in PLWHIV and NHP^43,44^. Collectively, our data indicate that SIV infection did not drastically impair the generation of mycobacterial-specific T cell or antibody responses in airways and blood. We previously reported an influx of mycobacterial-specific CD4^+^ and CD8^+^ T cells in the airways of IV BCG-vaccinated SIV-naive rhesus macaques, which correlated with protection^16,45^. Here, we observed a similar influx of airway CD4^+^ and CD8^+^ T cells, and increased circulating CD4^+^ T cell frequencies, in SIV+ and SIV-naive animals shortly after IV BCG vaccination. Polyfunctional T_H_17 T cells in airways following mucosal BCG vaccination have been correlated with protection^15^ and are associated with better Mtb control in granulomas^46^. We observed here an influx of IL-17A-producing CD4^+^ T cells in airways and an increase in circulating T_H_1/T_H_17 (T_H_1*) CD4^+^ T cell frequencies in both SIV+ and SIV-naive groups after IV BCG vaccination. Mycobacterial-specific antibodies, which have been associated with IV BCG efficacy and Mtb control^20,47^, increased in plasma and airways following IV BCG vaccination, regardless of SIV status.

It is important to note that the vaccinated animals were treated with anti-mycobacterial drugs beginning within 4 weeks of vaccination as an added precaution against disseminated BCG in SIV+ animals. Yet the IV BCG-induced immune responses and protection were not diminished but rather maintained in all SIV-naive macaques and most SIV+ macaques. These data suggest that BCG can be killed soon after vaccination without loss of protection. IV BCG has been shown to induce an influx of CD3^+^ T cells in lung tissue and airways and CD11c^+^ antigen-presenting cells in lung tissue 1 month after vaccination in SIV-naive rhesus macaques^16^. This early influx of cells to airways and lung, around the same time frame we initiated HRE, may indicate that sterilizing immune mechanisms are established within the first month after vaccination, although exact BCG kill kinetics were not measured in our study and it is possible that longer antigen exposure is required for durable protection.

We noted a transient spike in plasma viraemia 2 weeks after IV BCG vaccination, which was mirrored by an increased frequency of circulating CCR5^+^ CD4^+^ T cells 2 weeks later. BCG vaccination has been shown to increase these cells in both HIV-exposed infants and infant macaques^48,49^. Given that CCR5^+^ CD4^+^ T cells are a target of SIV^32^, the observed spike in viraemia is probably due to the expansion of infectable cells induced by BCG. This transient burst of SIV replication following IV BCG may increase the viral reservoirs in tissues and represent a safety signal for TB vaccines in the context of HIV infection. However, this effect may be mitigated by concurrent ART.

Three SIV+ vaccinated animals were unprotected from Mtb challenge. These animals tended to have a higher viral setpoint, more circulating T_FH_ and fewer CD4^+^ T cells in BAL, suggesting that unprotected animals had progressive SIV infections^30,50,51^. In a study of BCG co-infection of SIV+ rhesus macaques, animals with high plasma viraemia (>10^6^ copies ml^−1^) exhibited fewer circulating CD4^+^ T cells and disseminated BCG^52^. Airways were not sampled in that study. While we did not observe disseminated BCG in the three unprotected animals, it is possible that the progressive SIV infection ablated the development of an effective anti-mycobacterial T cell response elicited by IV BCG. Conversely, the SIV+ animals that were protected by IV BCG tended to have more CD4^+^ T cells in the airways and better-controlled SIV.

There are several limitations to this study. SIV infection in MCM was quasi-pathogenic: some SIV+ vaccinated animals were able to naturally control SIV replication, while others (*n* = 2) maintained high plasma viraemia (>10^6^ copies ml^−1^). While we do believe there is some level of immune defect given that, as shown previously^22^ as well as here, unvaccinated SIV+ MCM co-infected with Mtb have a higher bacterial burden regardless of viraemic control compared with animals infected with Mtb alone, we recognize that animals with better-controlled SIV plasma viraemia were completely protected. This may highlight the need for viraemic control, achieved either naturally or by ART, to ensure IV BCG-elicited sterilizing immunity in SIV+ animals. The link between sterilizing immunity and viraemic control will be explored in future studies. The 4-month vaccination period, chosen to minimize possible confounding variables introduced by SIV pathogenesis (for example, lymphoma, thrombocytopaenia and so on), did not assess vaccine durability and sterilizing immunity, which may be due to, in part, short-lived changes in innate immune cells. BCG has been shown to enhance innate immunity against TB through elevated cytokine production of monocytes and epigenetic modifications of haematopoietic stem cells, referred to as trained immunity^53–56^. The effect of BCG on trained immunity has been demonstrated to last 3 months to 1 year after IV vaccination in healthy volunteers^57,58^. While previous data suggest that trained immunity may not occur with IV BCG in SIV-naive rhesus macaques (that is, no culturable BCG from the bone marrow and no increase in innate activation to non-specific stimuli)^16^, this aspect of immunity was not investigated in the current study. It is possible that protection may wane due to the loss of innate immune cell functions over time.

There are still many unknowns about sterilizing immunity generated by IV BCG in SIV+ macaques. First, it is not yet known precisely how long BCG needs to be alive to elicit protection. We do not know the exact kill kinetics of HRE in our system and, therefore, the timing of when BCG is fully cleared. A self-limiting auxotrophic BCG strain may represent an effective alternative without the need for a post-vaccination antibiotic regimen^59^. Second, the durability of IV BCG-elicited protection is unknown, especially in an immunocompromised host. Studies with longer vaccine intervals are needed to determine durability of protection and immune correlates associated with long-lived immunity. Third, alternative routes of administration, such as mucosally delivered BCG^15,60^ or MTBVAC^54,61^, have yet to be explored in SIV-infected animals. Overall, this study shows robust vaccine-elicited protection against Mtb infection and disease using a NHP model of HIV. Furthermore, this study establishes a model to identify correlates of protection in the context of pre-existing SIV/HIV and lays the groundwork for future studies to develop effective TB vaccine regimens for PLWHIV.

## Methods

### Animals

Adult Mauritian cynomolgus macaques (*Macaca fascicularis*; age >4 years old; *n* = 40) were obtained from Bioculture US (Extended Data Table 1). MHC haplotype was determined by MiSeq sequencing and animals with the presence of at least one copy of the M1 MHC haplotype were selected for this study^62,63^.

Animal protocols and procedures were approved by the University of Pittsburgh Institutional Animal Care and Use Committee, which adheres to guidelines established in the Animal Welfare Act and the Guide for the Care and Use of Laboratory Animals, as well as the Weatherall Report (8th Edition). The University is fully accredited by the Association for Assessment and Accreditation of Laboratory Animal Care International (accreditation number 000496), and its Office of Laboratory Animal Welfare assurance number is D16-00118. The Institutional Animal Care and Use Committee reviewed and approved the study protocols 15035407 and 18032418, under assurance numbers A3187-01 and D16-00118, respectively.

Animal welfare was monitored twice daily for overall physical health (weight, appetite, activity level, and so on) as described previously^23^. Animals were monitored closely following Mtb challenge for clinical signs of TB (for example, weight loss, tachypnoea, dyspnoea or coughing). In addition, regular PET/CT imaging was conducted to monitor TB progression. Animals were sedated for all veterinary procedures (for example, blood draws) using ketamine or other approved drugs. Veterinary technicians monitored animals especially closely for any signs of pain or distress and provided appropriate supportive care (for example, dietary supplementation and rehydration) and treatments (analgesics) when necessary. Any animal considered to have advanced disease or intractable pain from any cause, was deemed to have reached the humane endpoint, sedated with ketamine and humanely euthanized using sodium pentobarbital.

### SIV infection

Vaccinated and unvaccinated macaques designated for SIV infection were infected intrarectally with SIVmac239 (3,000 50% tissue culture infectious dose IU). Plasma viraemia was monitored serially by quanititative PCR (qPCR) as previous described^64,65^. Viral RNA was isolated using the Maxwell Viral Total Nucleic Acid Purification Kit (Promega) and reversed transcribed using the TaqMan Fast Virus 1-Step quantitative reverse transcription PCR (qRT–PCR) Kit (Invitrogen). DNA was quantified on a LightCycler 480 (Roche).

### BCG vaccination

BCG Danish Strain 1331 (Statens Serum Institute) was expanded (by Aeras, now AVI) and frozen at approximately, 3 × 10^8^ CFU ml^−1^. Just before BCG injection, BCG was thawed and diluted in sterile PBS to approximately 5 × 10^7^ CFU ml^−1^ (actual: 8 × 10^7^ CFU ml^−1^). IV BCG was injected into the left saphenous vein in a volume of 1 ml. An aliquot of inoculum was plated on 7H11 agar and CFU was enumerated 3 weeks later to ensure an accurate input inoculum.

### HRE administration

Isoniazid (Teva Pharmaceuticals; 15 mg kg^−1^, per os), rifampin (Darmerica; 20 mg kg^−1^, per os) and ethambutol (Lupin Pharmaceuticals; 55 mg kg^−1^, p.o.), referred to together as HRE, obtained from The Pet Apothecary, was administered once daily for 8 weeks starting 3–4 weeks after IV BCG vaccination. Unvaccinated animals received HRE to minimize any confounding effects due to antibiotic therapy.

### Mtb challenge

All animals were infected with a low dose (4–21 CFU) of Mtb Erdman via bronchoscopic instillation, as described previously^21^.

### Clinical and microbiological monitoring

All animals were assessed twice daily for general health over the entirety of the study. Blood cultures were performed 2 weeks after IV BCG to assess BCG CFU ml^−1^. Blood was plated on two 7H11 plates at 500 μl per plate, incubated for 21 days and colonies were enumerated. Following Mtb challenge, animals were monitored closely for clinical signs of TB (coughing, weight loss, tachypnoea, dyspnoea and so on) following Mtb challenge. Monthly gastric aspirates and bronchoalveolar lavage samples were tested for Mtb growth. Blood was drawn at regular intervals to measure erythrocyte sedimentation rate and to provide PBMC and plasma.

### PBMC and BAL processing

PBMC were isolated from blood using Ficoll-Paque PLUS gradient separation (GE Healthcare Biosciences). Single-cell suspensions were cryopreserved in foetal bovine serum containing 10% dimethylsulfoxide in liquid nitrogen. BAL wash fluid (4 × 10 ml washes of PBS) was pelleted and an aliquot of wash fluid (15 ml) was cryopreserved. The remaining cells were resuspended into ELISpot medium (Roswell Park Memorial Institute medium 1640, 10% heat-inactivated human albumin, 1% l-glutamine and 1% 4-(2-hydroxyethyl)-1-piperazineethanesulfonic acid) and counted. BAL cells were then divided into their appropriate flow cytometry assay depending on cell yield.

### Multiparameter flow cytometry

Longitudinal BAL and PBMC samples were stained for leukocyte composition (phenotype) or antigen-specific T cell responses. In general, BAL cells were stained immediately and PBMC were cryopreserved and batch-analysed at the end of the study.

For BAL analyses, cells were counted and aliquoted into a 96-well plate for either phenotype or intracellular cytokine responses (1 × 10^6^ cells per well). In cases where total BAL cell counts were low, cells were prioritized for the 14-h stimulation assay. For the phenotype panel, cells were reconstituted in 500 nM dasatinib in ELISpot media to improve MHC class I-related protein (MR1) (5-OP-RU; BV421) tetramer staining. Cells were incubated with MR1 tetramer (National Institute of Health (NIH) Tetramer Core Facility) for 30 min at 4 °C. The MR1 technology was developed jointly by J. McCluskey, J. Rossjohn and D. Fairlie, and the material was produced by the NIH Tetramer Core Facility as permitted to be distributed by the University of Melbourne. Cells were then washed and stained for viability (Zombie Live/Dead near IR; Invitrogen) for 10 min at room temperature (25 °C). Cells were then washed, incubated with surface antibody cocktail for 20 min at 4 °C, and fixed with 1% paraformaldehyde for 15 min. For the intracellular cytokine assay, cells were aliquoted (1 × 10^6^ cells per well) into either a media control well, PPD well (20 μg ml^−1^; AJ Vaccines) or ESAT-6/CFP-10 overlapping peptide pools (1 μg ml^−1^ each; BEI Resources). Stimulators were added and incubated for 2 h, then brefeldin A (1 μg ml^−1^; BioLegend, cat. no. 420601) and monensin (1 μg ml^−1^; BioLegend, cat. no. 420701) was added for the remainder of the stimulation time (12 h). Cells were washed and surface markers were stained similarly as described for the phenotype panel. However, after paraformaldehyde fixation, cells were permeabilized with BD Cytofix/Cytoperm (BD, cat. no. 554714) for 10 min at room temperature. Intracellular markers were stained for 20 min at room temperature. Stained BAL cells were run within 2 days of staining.

Flow cytometry of BAL was performed using a Cytek Aurora (BD, SpectroFlo v2.2). Flow cytometry standard files were analysed using FlowJo software for Macintosh (version 10.1). Frequencies relative to live leukocytes were used to calculate cell counts per BAL collection (Fig. 2). In brief, cell counts enumerated on a haemocytometer were multiplied by the frequency of the cell population of interest (for example, T cells) relative to live leukocytes. BAL samples with low viability (<70%) or elevated background autofluorescence were excluded from analyses. Lastly, due to the coronavirus disease 2019 pandemic, several BAL collections were not collected to ensure the health and safety of our technical staff. Therefore, the number of BAL samples indicated in Fig. 2 are reflective of samples collected before and over the course of the coronavirus disease 2019 pandemic and met the inclusion criteria described above. Individual animal data are shown in Supplementary Data 1.

For PBMC analyses, cells were divided to be stained for phenotype or 14-h stimulation as previously described^16^. PBMC stimulators include: H37Rv whole cell lysate (20 μg ml^−1^; BEI Resources) and ESAT-6/CFP-10 peptide pools (1 μg ml^−1^ each). Cells were stained as follows: cells were stained with MR1 tetramer for 20 min, washed twice with PBS/bovine serum albumin (BSA) (0.1%), then stained with viability dye for 20 min, washed twice with PBS/BSA (0.1%) and incubated with human fragment crystallizable receptor blocking reagent (Miltenyi); surface markers were stained for 20 min and washed three times with PBS/BSA (0.1%); cells were permeabilized with BD Cytofix/Cytoperm (BD, cat. no. 554714) for 20 min; and intracellular markers were stained for 30 min. Data were acquired on a modified BD LSR Fortessa (FACS Diva v9.3) and analysed in FlowJo software for Macintosh (version 10.8.1). Gating strategies are shown in Supplementary Data 1–4. All cytokine data presented are background-subtracted. Antibodies used in BAL and PBMC panels are listed in Supplementary Table 1.

### ELISPOT

ELISPOT was performed as previously described^16^. In brief, 96-well plates were coated with monkey IFNγ antibody (clone MT126L, 15 μg ml^−1^), incubated overnight at 4 °C and wells were washed with sterile PBS. Wells were then blocked for 2 h at 37 °C at 5% CO_2_ with ELISpot medium (Roswell Park Memorial Institute medium 1640 + 10% human albumin + 1% l-glutamine + 1% 4-(2-hydroxyethyl)-1-piperazineethanesulfonic acid) and washed with sterile PBS. Frozen PBMC were thawed and enumerated by haemocytometer. Cells were aliquoted into wells at 2 × 10^5^ cells per well (150 μl). Stimulators or ELISPOT media control (50 μl) were added to the appropriate wells, and plates were incubated at 37 °C at 5% CO_2_ for 40–48 h. The stimulators and the final concentrations used in this assay include: CFP peptide pools (2 μg ml^−1^), ESAT-6 peptide pools (2 μg ml^−1^), CFP-10 peptide pools (2 μg ml^−1^) and phorbol 12,13-diburtyrate and ionomycin (12.5 μg ml^−1^ and 37.5 μg ml^−1^, respectively). Wells were washed with PBS and incubated with biotinylated anti-human IFNγ antibody (clone 7-B6, 2.5 μg ml^−1^) for 2 h at 37 °C at 5% CO_2_. Wells were again washed with PBS and incubated this time with streptavidin-linked horseradish peroxidase (1:100 dilution in PBS + 0.5% foetal bovine serum) for 45 min at 37 °C at 5% CO_2_. After washing with PBS for a third time, 3-amino-9-ethylcarbazole substrate was added to the wells and the plate was developed for 5–8 min in the dark (3-amino-9-ethylcarbazole kit). Finally, wells were washed with diH_2_O, fixed with 2% PFA for 10 min, washed with PBS and dried overnight. Spot forming units were counted manually on an ELISpot reader (ImmunoSpot v5.1).

### Serology

The antibody ELISAs were performed as previously described by Darrah et al. 2020 (ref. ^16^). MaxiSorp ELISA plates (96 wells; Thermo Scientific) were coated with 100 μl of H37Rv WCL at a concentration of 1 μg ml^−1^ per well at 4 °C overnight. The coated plates were blocked with 100 μl of 1× blocking solution (PBS + 10% FBS) for 2 h at room temperature. Plates were washed six times with PBS-Tween between each step. Plasma or 10× BAL concentrate from each animal were 1:5 serially diluted. A volume of 100 μl was added and incubated at 37 °C for 2 h. After 2 h, plates were incubated with 100 μl of diluted horseradish peroxidase-conjugated antibody. Plates were incubated for 1 h at room temperature. A final wash step was done, samples were incubated for approximately 12 min with 100 μl Ultra TMB ELISA substrate (Invitrogen, cat. no. 34029). The reaction was stopped by adding 100 μl of 2N sulfuric acid. The plates were read with a Promega Glomax Multi detection system (v1.3.2) at 450 nm. Analysis was done using GraphPad Prism software (version 8.2.1). Data are presented as area under the curve (AUC).

### PET/CT

Radiolabeled 2-deoxy-2-(^18^F)fluoro-d-glucose (FDG) PET/CT imaging was performed 4, 8 and 12 weeks after Mtb challenge. Imaging was performed using a MultiScan LFER-150 PET/CT scanner (Mediso Medical Imaging Systems) housed within our biosafety level 3 facility as previously described^66,67^. Co-registered PET/CT images were analysed using OsiriX MD software (version 12.5.2, Pixmeo) to enumerate granulomas and to calculate the total FDG avidity of the lungs, exclusive of lymph nodes, which are a quantitative measure of total inflammation in the lungs^66,68^. For historical controls, PET/CT scans were performed using a microPET Focus 220 preclinical PET scanner (Siemens Molecular Solutions) and a clinical eight-slice helical CT scanner (NeuroLogica Corporation)^68^. Thoracic lymphadenopathy and extrapulmonary dissemination of Mtb to the spleen and/or liver were also assessed qualitatively on these scans.

### Necropsy, pathology and bacterial load

Necropsies were performed at around 12 weeks following Mtb challenge as previously described^22,23^ We used an established scoring system to quantitatively assess gross pathology^21^. Pathology scores were calculated and reflect overall TB disease burden for each animal. Tissue samples were divided and a portion were fixed in 10% neutral-buffered formalin for histopathology; the remainder were homogenized to a single-cell suspension as described previously^21^. Serial dilutions of these homogenates were plated onto 7H11 agar and incubated at 37 °C at 5% CO_2_ for 3 weeks, and colonies were enumerated. Bacterial burden in lungs, thoracic lymph nodes, liver and spleen, as well as total thoracic CFU, were calculated as described previously^21^. Neutral-buffered formalin-fixed tissue was embedded in paraffin, sectioned and stained with haematoxylin and eosin for histopathologic examination.

### Statistics including power analysis

We used total thoracic bacterial burden (log_10_) as the primary outcome variable with a pooled standard deviation (s.d.) of 1.09 (calculated by averaging s.d. of all groups) for a two-sided test and adjusted the type I error for two comparisons (*α* = 0.025). For the unvaccinated (*n* = 8) and IV BCG (*n* = 7) comparison, we obtained 83.0% power to detect a mean difference of 2 in bacterial burden. For the SIV/Unvaccinated (n = 11) and SIV/IV BCG (n = 12) comparison, we obtained 97.0% power to detect a mean difference of 2. Normality was tested by using Shapiro–Wilk test. Significance of plasma viraemia relative to BCG within SIV+ groups was assessed using repeated measure one-way analyses of variance (ANOVAs) paired with Dunnett’s multiple comparisons tests to assess significance relative to ‘pre-BCG’. For all other longitudinal comparisons between vaccinated groups, linear mixed effects models with subject as a random variable were used (Supplementary Table 2). Fixed effect tests were used to assess differences among timepoints and vaccine groups (including an interaction term of time × vaccine). Timepoints were compared with ‘pre-BCG’ control using Dunnett’s multiple comparison tests. When a treatment effect was present (that is, SIV infection), we performed Mann–Whitney tests at individual timepoints (not adjusting for multiple comparisons). For comparisons among all four groups across specific timepoints (for example, pre- versus post-Mtb), linear mixed effects models with Šidák’s multiple comparisons were used (Supplementary Table 2). Comparisons between pre and post (relative to either BCG or Mtb challenge) were tested using either paired *t*-tests or Wilcoxon paired signed rank tests, depending on normality. Kruskal–Wallis tests were performed with Dunn’s multiple comparisons between SIV-naive, vaccinated and unvaccinated groups, and SIV+, vaccinated and unvaccinated groups, for necropsy outcome data (for example, lung inflammation, TB pathology and Mtb burden). Statistical tests were performed in Prism (version 8.2.1; GraphPad). All tests were two-sided and statistical significance was designated at a *P* value of <0.05.

### Reporting summary

Further information on research design is available in the Nature Portfolio Reporting Summary linked to this article.

### Supplementary information

Supplementary information Supplementary Data 1–8.

Reporting summary

Supplementary table Supplementary Table 1. List of antibodies by flow panel. Supplementary Table 2. Mixed model statistics of BAL and PBMC composition and responses during IV BCG vaccination and Mtb challenge.

### Source data

Source Data Fig. 1 Source data for Fig. 1.

Source Data Fig. 2 Source data for Fig. 2.

Source Data Fig. 3 Source data for Fig. 3.

Source Data Fig. 4 Source data for Fig. 4.

Source Data Fig. 5 Source data for Fig. 5.

Source Data Fig. 6 Source data for Fig. 6.

Source Data Extended Data Fig./Table 1 Source data for Extended Data Fig. 1.

Source Data Extended Data Fig./Table 2 Source data for Extended Data Fig. 2.

Source Data Extended Data Fig./Table 3 Source data for Extended Data Fig. 3.

Source Data Extended Data Fig./Table 4 Source data for Extended Data Fig. 4.

Source Data Extended Data Fig./Table 5 Source data for Extended Data Fig. 5.

Source Data Extended Data Fig./Table 6 Source data for Extended Data Fig. 6.

## Figures and Tables

**Extended Data Table 1:**
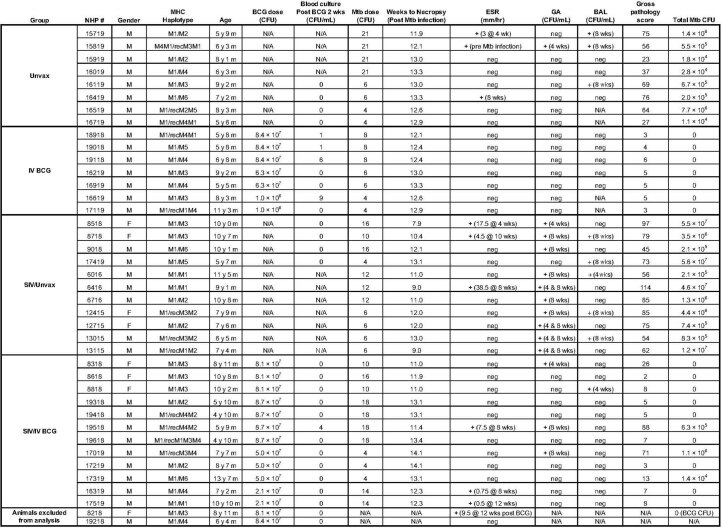
Summary of MCM and outcome measures following IV BCG vaccination and Mtb challenge

Historical controls in the SIV/Unvax group include: 6016, 6416, 6716, 12415, 12715, 13015, and 13115. Age is indicated as years (y) and months (m). For ESR, GA, and BAL, time point indicated post Mtb infection, unless otherwise stated. Total Mtb CFU limit of detection (LOD) is < 100 CFU per animal. One haplotype from 15819 expressed the M3 class I A alleles, but M1 class I B and II alleles - the M3 and M1 class I A regions share the major MHC class I A allele Mafa-A1*063, but the signal peptide sequence differs by one amino acid between the two haplotypes54. The recombinant haplotype of 171-19 contains M1 MHC class I A alleles and M4 MHC class I B and class II alleles. The recombinant haplotype of 131-15 contains M1 MHC class I B alleles and M2 MHC class I A and class II alleles.

## Data Availability

All relevant data are available from the corresponding author upon reasonable request. Source data are provided with this paper.
