# Author Correction: Intravenous Bacille Calmette–Guérin vaccination protects simian immunodeficiency virus-infected macaques from tuberculosis

**DOI:** 10.1038/s41564-024-01631-y

**Published:** 2024-02-14

**Authors:** Erica C. Larson, Amy L. Ellis-Connell, Mark A. Rodgers, Abigail K. Gubernat, Janelle L. Gleim, Ryan V. Moriarty, Alexis J. Balgeman, Cassaundra L. Ameel, Solomon Jauro, Jaime A. Tomko, Kara B. Kracinovsky, Pauline Maiello, H. Jake Borish, Alexander G. White, Edwin Klein, Allison N. Bucsan, Patricia A. Darrah, Robert A. Seder, Mario Roederer, Philana Ling Lin, JoAnne L. Flynn, Shelby L. O’Connor, Charles A. Scanga

**Affiliations:** 1 Department of Microbiology and Molecular Genetics, School of Medicine, University of Pittsburgh, Pittsburgh, PA USA; 2 Center for Vaccine Research, School of Medicine, University of Pittsburgh, Pittsburgh, PA USA; 3 Department of Pathology and Laboratory Medicine, University of Wisconsin, Madison, WI USA; 4 Division of Laboratory Animal Resources, School of Medicine, University of Pittsburgh, Pittsburgh, PA USA; 5 Vaccine Research Center, National Institute of Allergy and Infectious Diseases, National Institutes of Health, Bethesda, MD USA; 6 Department of Pediatrics, Children’s Hospital of Pittsburgh, School of Medicine, University of Pittsburgh, Pittsburgh, PA USA; 7 Wisconsin National Primate Research Center, University of Wisconsin, Madison, WI USA

**Keywords:** Tuberculosis, HIV infections, Vaccines

Correction to: *Nature Microbiology* 10.1038/s41564-023-01503-x, published online 9 October 2023.

In the version of the article initially published, the first sentence of the Acknowledgements listed an incorrect grant number, and has been updated to read “These studies were supported in whole or in part with federal funds from the National Institute of Allergy and Infectious Diseases, National Institutes of Health, and the Department of Health and Human Services, under the following: the NIH R01 AI-111815 award, the NIAID Contract No. 75N93019C00071 (IMPAc-TB), and the Intramural Research Program of the NIH/NIAID (VRC).” This has been corrected in the HTML and PDF versions of the article.

