# Supplementary material for: Intravenous Bacille Calmette–Guérin vaccination protects simian immunodeficiency virus-infected macaques from tuberculosis
Source: Nat Microbiol. 2023 Oct 9;8(11):2080–92. doi: 10.1038/s41564-023-01503-x (PMC10627825; doi:10.1038/s41564-023-01503-x)
Supplement: Supplementary file 1 — Supplementary Data 1–8. [file 41564_2023_1503_MOESM1_ESM.pdf]

nature microbiology

Article

https://doi.org/10.1038/s41564-023-01503-x

Intravenous Bacille Calmette–Guérin vaccination protects simian immunodeficiency virus-infected macaques from tuberculosis

In the format provided by the authors and unedited

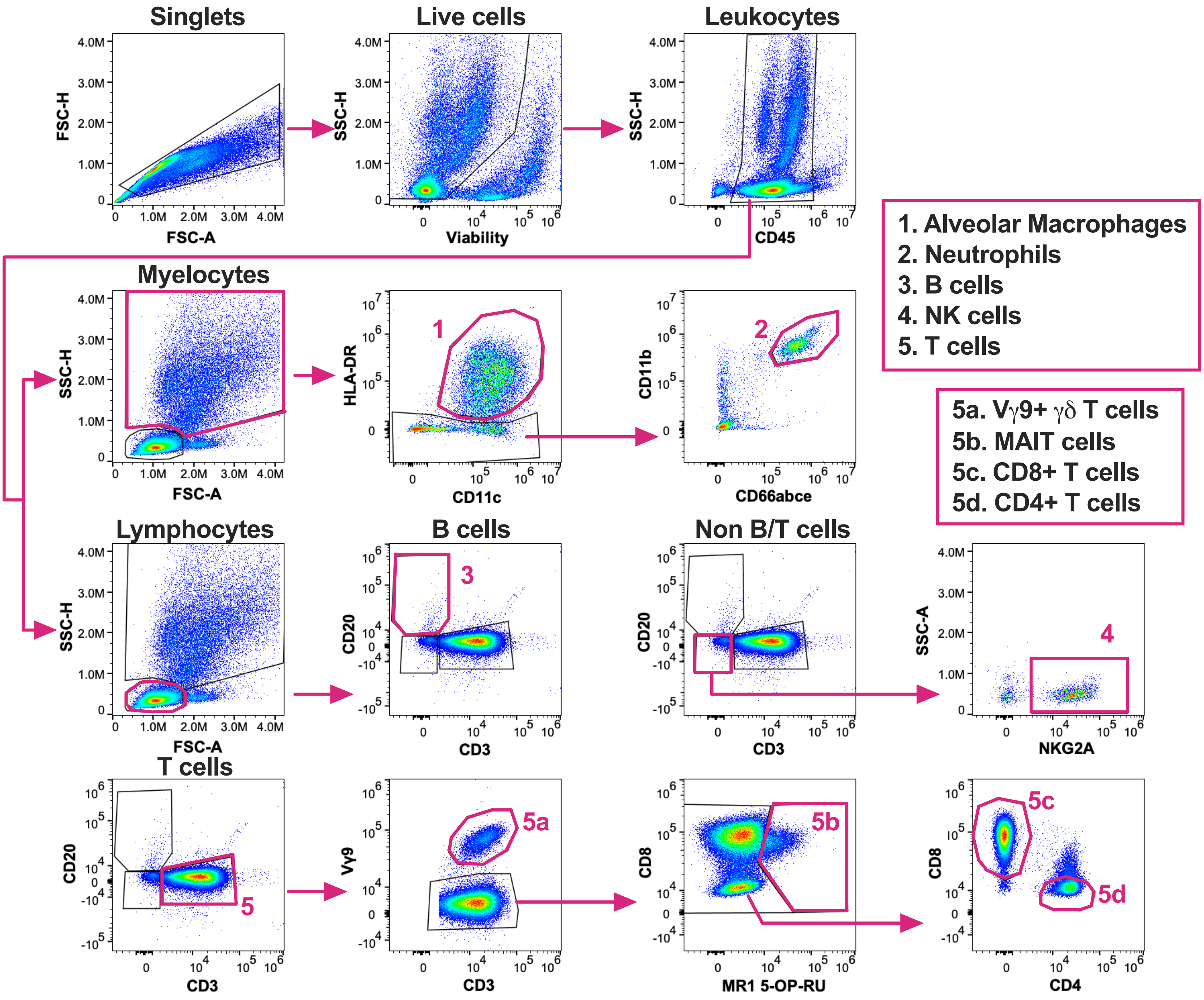

Supplementary Data 1. Gating schematic for BAL Phenotype panel. Fresh BAL cells were stained for flow cytometric analysis as indicated in the Methods and Supplementary Table 1, to determine the leukocyte frequencies and phenotypes prior to and after IV BCG vaccination. Total BAL were gated for singlets, followed by gating for live cells and then leukocytes (CD45 vs. SSC-H). Leukocytes were gated into two populations: myelocytes and lymphocytes. From the myelocyte gate, alveolar macrophages (CD11c+HLADR+) and neutrophils (HLADR-CD66abce+CD11b+) were characterized. From the lymphocyte gate, three subpopulations were gated based on CD20 and CD3 expression: B cells (CD20+CD3-), Non-B/T cells (CD20-CD3-), and T cells (CD20-CD3+). Non-B/T cells were further characterized into NK cells (NKG2A+). T cells (CD20-CD3+ cells) were further categorized as Vγ9+ γδ T cells, then MAIT cells (MR1 5-OP-RU tetramer+), then CD4+ or CD8+ T cells.

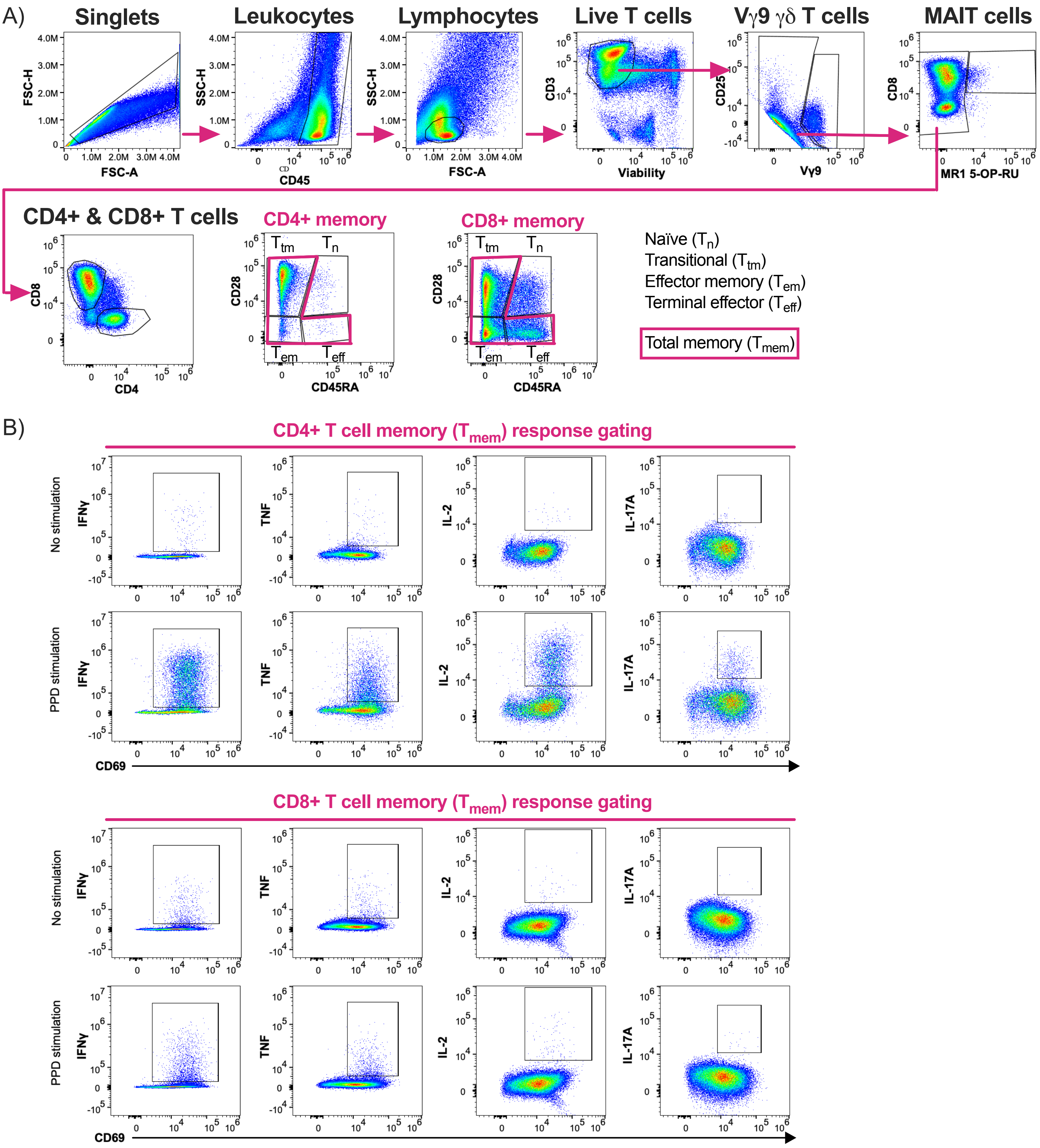

Supplementary Data 2. Gating schematic for BAL ICS panel. Fresh BAL cells were stained for flow cytometric analysis as indicated in the Methods and Supplementary Table 1, to determine CD4+ and CD8+ T cell memory responses to PPD (14h stimulation) prior to and after IV BCG vaccination. A) Total BAL were gated for singlets, followed by gating for leukocytes (CD45 vs. SSC-H), then lymphocytes, and then live T cells (CD3+). T cell subsets were characterized by Vγ9+ γδ T cells, then MAIT cells (MR1 5-OP-RU tetramer+), then CD4+ or CD8+ T cells. T cell memory of CD4+ and CD8+ cells was characterized: Naïve T cells (T_n, CD45RA+CD28+); Transitional memory T cells (T_{tm}, CD45RA-CD28+); Effector memory T cells (T_{em}, CD45RA-CD28-); and Terminal effector T cells (T_{eff}, CD45RA+CD28-). Total memory (T_{mem}) was generated by OR gating of T_{tm}, T_{em}, and T_{eff}. B) CD4+ and CD8+ T cell memory (T_{mem}) response gating for IFNγ, TNF, IL-2, and IL-17A in the presence of no stimulation (media only) or PPD.

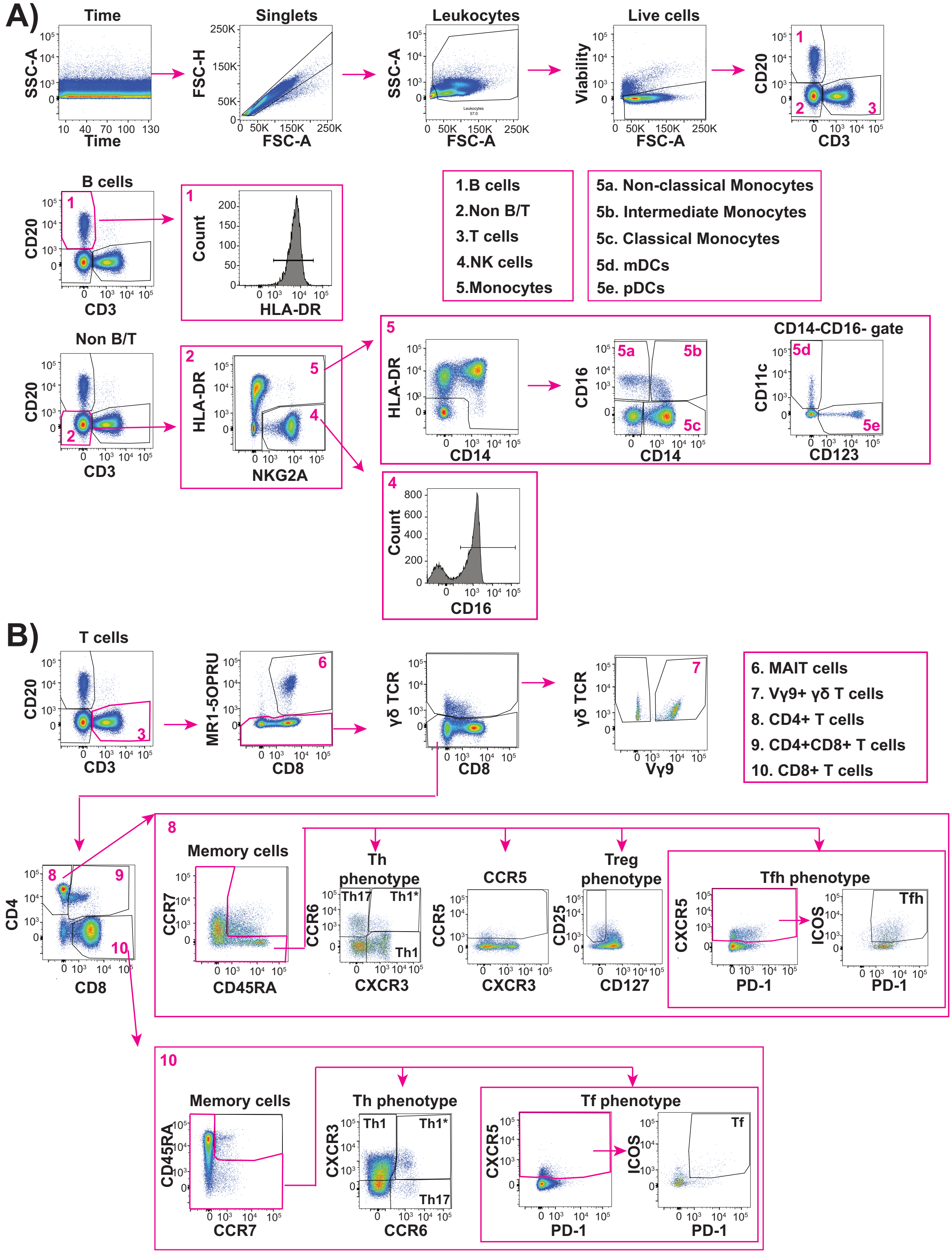

Supplementary Data 3. Gating schematic for PBMC Phenotype panel. Frozen PBMC were stained for flow cytometric analysis as indicated in the Methods and Supplementary Table 1, to determine the leukocyte frequencies and phenotypes prior to and after IV BCG vaccination and Mtb challenge. A) Total PBMC were gated for singlets, followed by gating for leukocytes (FSC-A vs. SSC-A). Live cells were gated into three subpopulations based on CD20 and CD3 expression: B cells (CD20+CD3-), Non-B/T cells (CD20-CD3-), and T cells (CD20-CD3+). Non-B/T cells were further characterized into NK cells (NKG2A+HLADR-CD16+ cells), Non-classical monocytes (HLADR+CD16+CD14-), Intermediate monocytes (HLADR+CD16+CD14+) or classical monocytes (HLADR+CD16-CD14+). Classical monocytes were sub-categorized as either myeloid dendritic cells (mDCs, CD11c+CD123-) or plasmacytoid dendritic cells (pDCs; CD11c-CD123+). B) T cells (CD20-CD3+ cells) were further categorized as MAIT cells (CD8+MR1 5OPRU tetramer+), then gamma delta T cells (γδ T-cell receptor+ (TCR+) cells) were separated from αβ T cells (γδ TCR- cells). γδ TCR+ cells were gated for expression of Vγ9 T-cell receptor (Vγ9- and Vγ9+ cells). γδ TCR- cells were categorized as CD4+, CD4+CD8+, or CD8+ T cells. Both CD4+ and CD8+ conventional T cells were characterized for memory phenotype (T_{mem}, cells that did not express the naïve CCR7+CD45RA+ phenotype), then memory cells were characterized for the expression of CXCR3 and/or CCR6 (T helper (Th) phenotypes), CCR5, and regulatory (T_{reg}; CD25+CD127-) or T follicular helper (T_{fh}) phenotype (CXCR5+ICOS+PD1+).

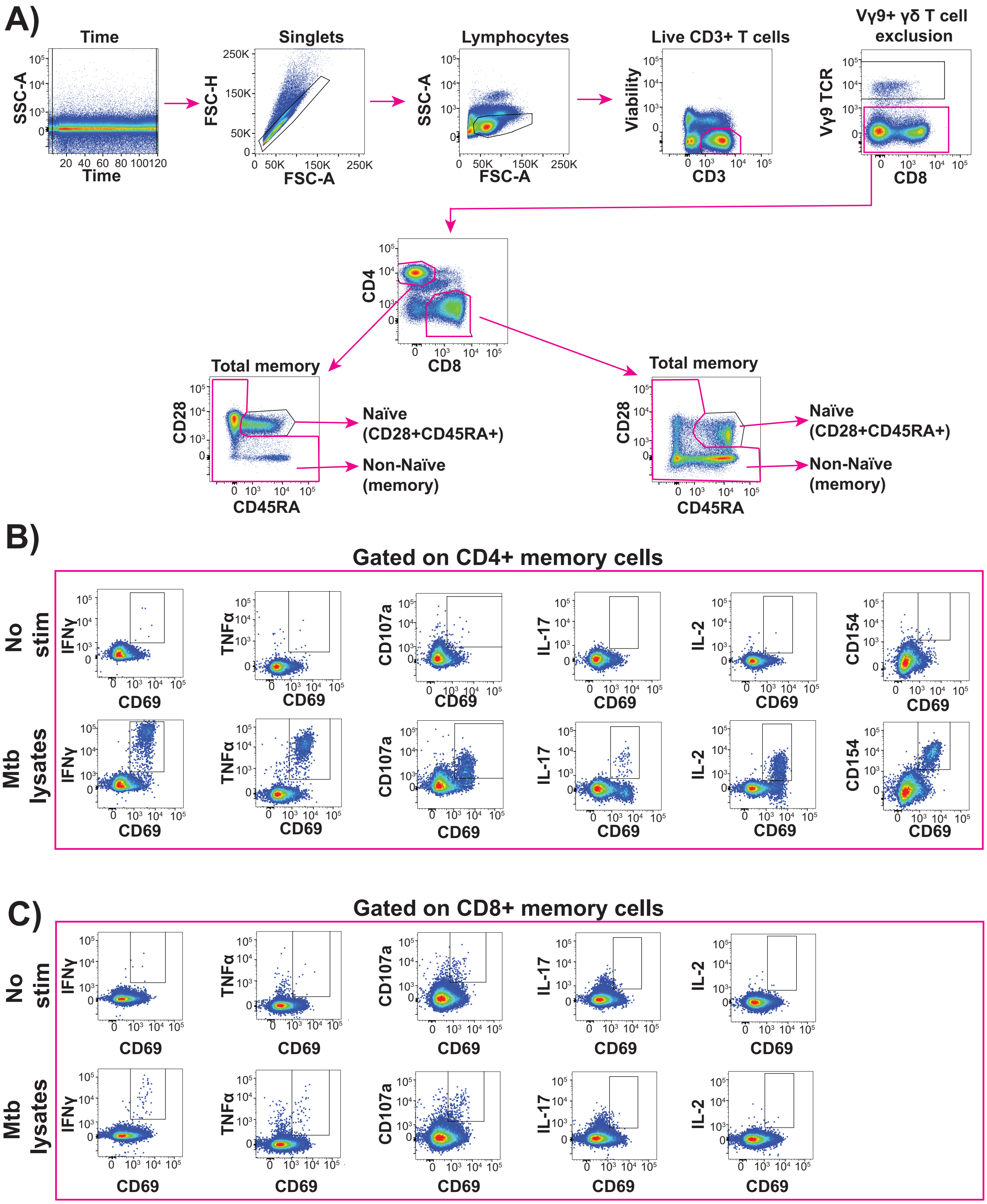

Supplementary Data 4. Gating schematic for PBMC ICS panel. Frozen PBMC were stained for flow cytometric analysis as indicated in the Methods and Supplementary Table 1, to determine CD4+ and CD8+ T cell memory responses to Mtb lysate and ESAT-6/CFP-10 (14h stimulation) prior to and after IV BCG vaccination and Mtb challenge. A) Total PBMC were gated for singlets, followed by gating for lymphocytes (FSC-A vs. SSC-A) and then live T cells (CD3+). Vγ9+ γδ T cells were excluded and the Vγ9- population was then sub-gated by CD4+ and CD8+ T cells. T cell memory of CD4+ and CD8+ cells was characterized by: Memory (T_{mem}; CD28+CD45RA-, CD28-CD45RA-, and CD28-CD45RA+) and Naïve (CD28+CD45RA+). B) CD4+ and CD8+ memory (T_{mem}) responses were gated by cytokine (IFNγ, TNF, IL-2, IL17A) or other markers (CD107a and CD154) versus CD69 in the presence and absence of no stimulation (media only) or Mtb lysate.

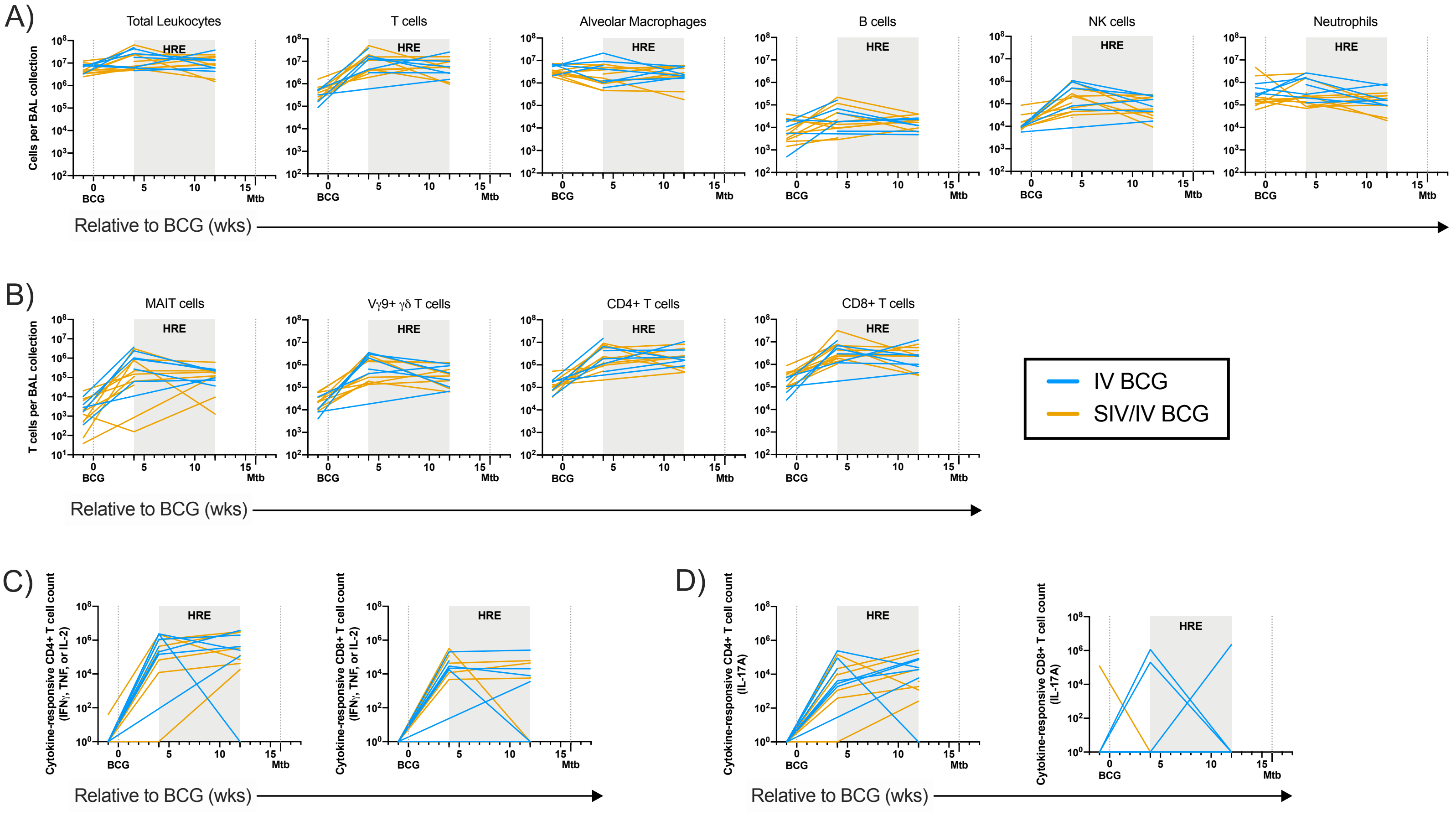

Supplementary Data 5. Leukocyte and T cell subsets and T cell response in BAL after BCG vaccination in individual animals. A) Number of leukocytes per BAL collection. B) Number of T cell subset cells per BAL collection. C) Number of cytokine-responsive (IFN, TNF, IL-2) CD4+ and CD8+ T cells in BAL after 14h stimulation with PPD. D) Number of IL-17A-responsive CD4+ and CD8+ T cells in BAL after 14h stimulation with PPD. A-D) Lines indicate individual animals of SIV-naïve (light blue) and SIV+ (gold) vaccinated groups. SIV-naïve vaccinated animals (IV BCG): pre BCG (n = 5), 4 wks post BCG (n = 6), and 12 wks post BCG (n = 6). SIV+ vaccinated animals (SIV/IV BCG): pre BCG (n = 10), 4 wks post BCG (n = 11), and 12 wks post BCG (n = 8).

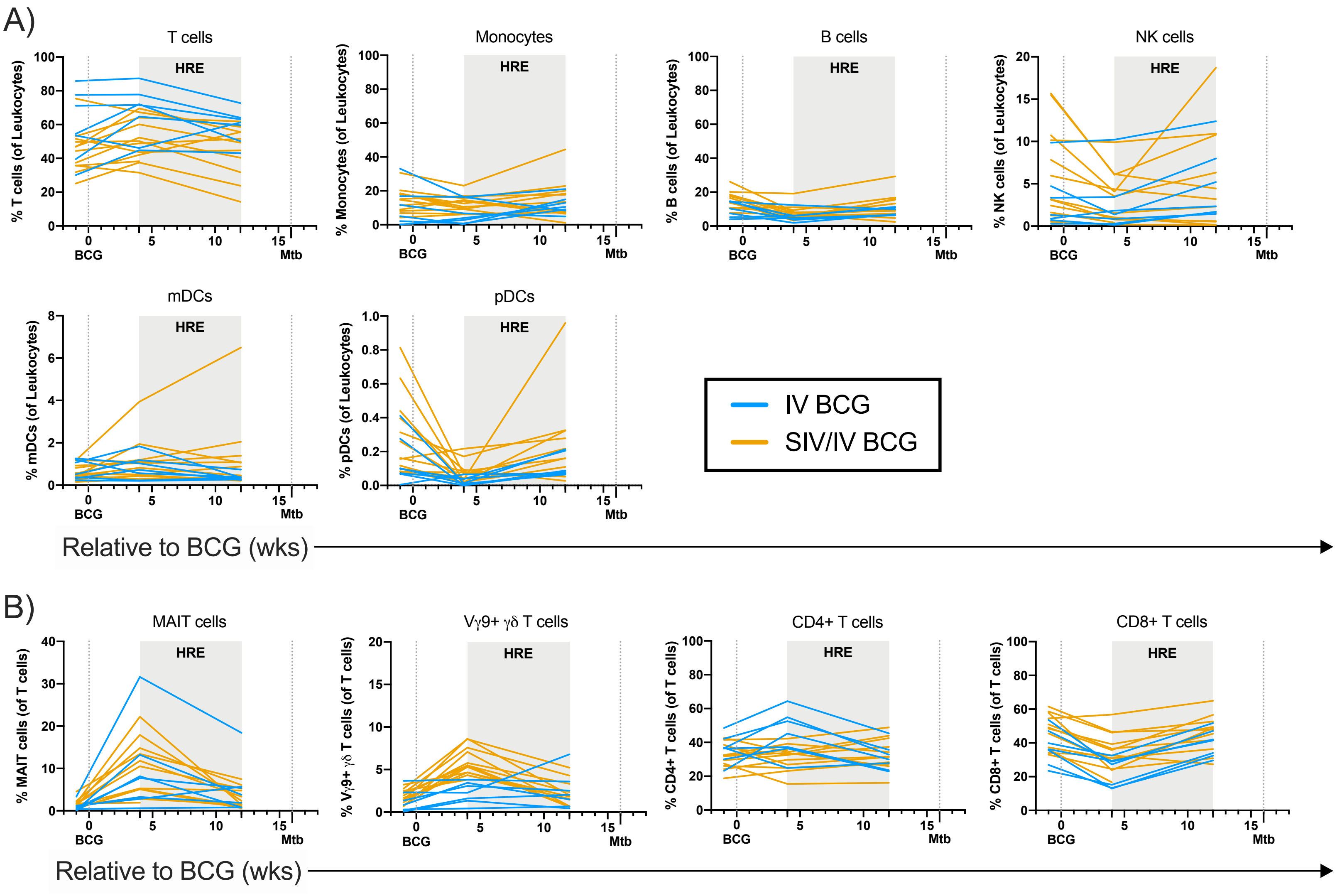

Supplementary Data 6. Frequencies of Leukocyte and T cell subsets in PBMC after vaccination in individual animals. A) Frequencies of leukocyte subsets relative to BCG. B) Frequencies of T cells and T cell subsets (MAIT, V9+, CD4+, and CD8+ T cells) relative to BCG. Lines indicate individual animals of SIV-naïve (IV BCG; light blue) and SIV+ (SIV/IV BCG; gold) vaccinated groups. SIV-naïve vaccinated animals: pre BCG (n = 7), 4 wks post BCG (n = 7), and 12 wks post BCG (n = 7). SIV+ vaccinated animals: pre BCG (n = 12), 4 wks post BCG (n = 12), and 12 wks post BCG (n = 11).

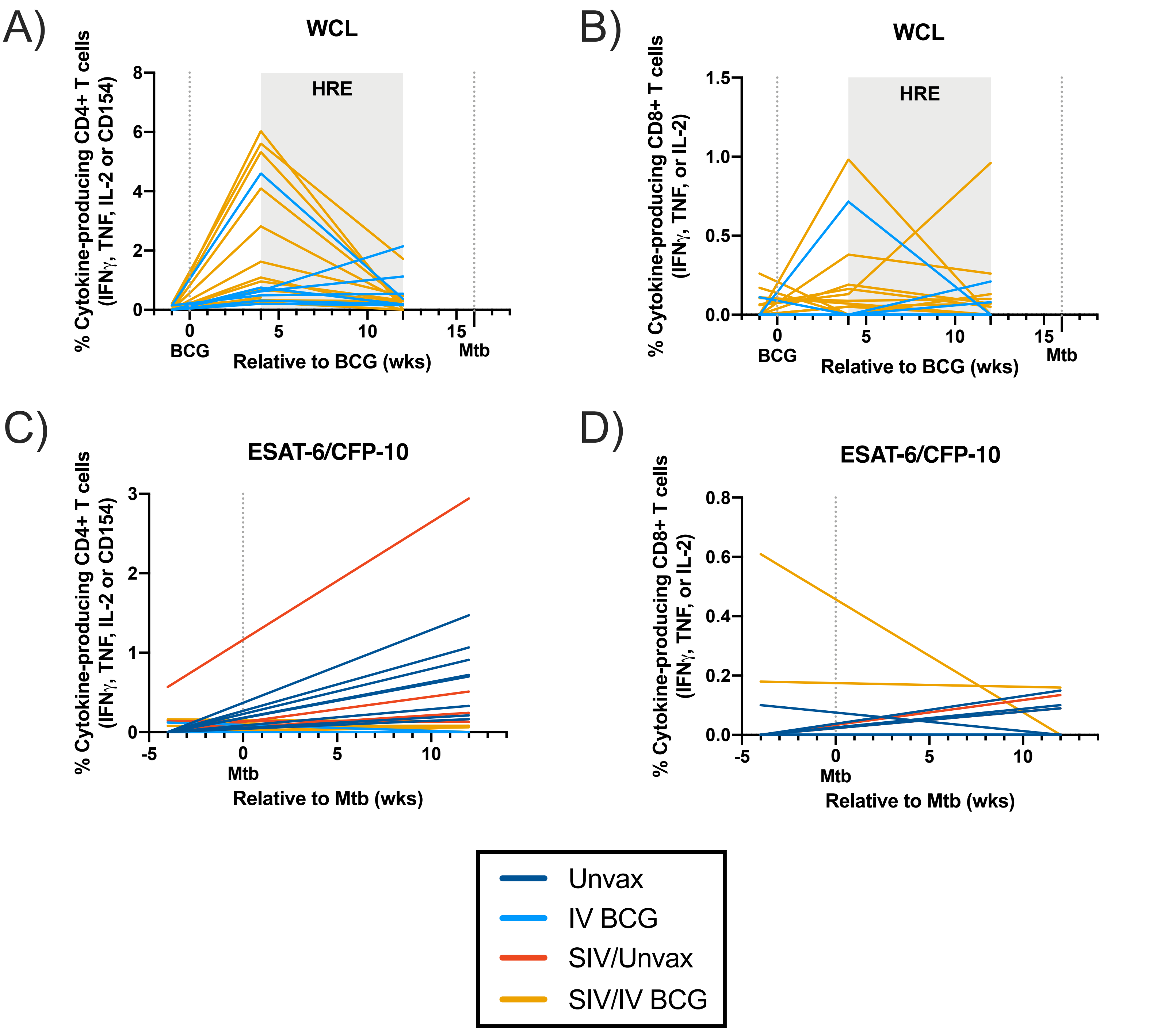

Supplementary Data 7. T cell response in PBMC after BCG vaccination and Mtb challenge in individual animals. A & B) Frequency of cytokine-responsive CD4+ (A) and CD8+ (B) T cells in PBMC after 14h stimulation with H37Rv whole cell lysate relative to BCG vaccination. C & D) Frequency of cytokine-responsive CD4+ (C) and CD8+ (D) T cells in PBMC after 14h stimulation with ESAT-6/CFP-10 relative to Mtb challenge. A-D) Lines indicate individual animals of Unvax (dark blue), IV BCG (light blue), SIV/Unvax (red) and SIV/IV BCG (gold) groups. Unvaccinated animals (Unvax): Pre Mtb (n = 8) and 12 wks post Mtb (n = 8). SIV-naïve vaccinated animals (IV BCG): pre BCG (n = 7), 4 wks post BCG (n = 7), and 12 wks post BCG/Pre Mtb (n = 7), and 12 wks post Mtb (n = 7). SIV+ unvaccinated (SIV/Unvax): Pre Mtb (n = 4) and 12 wks post Mtb (n = 4). SIV+ vaccinated animals (SIV/IV BCG): pre BCG (n = 12), 4 wks post BCG (n = 12), 12 wks post BCG/Pre Mtb (n = 11), and 12 wks post Mtb (n = 12).

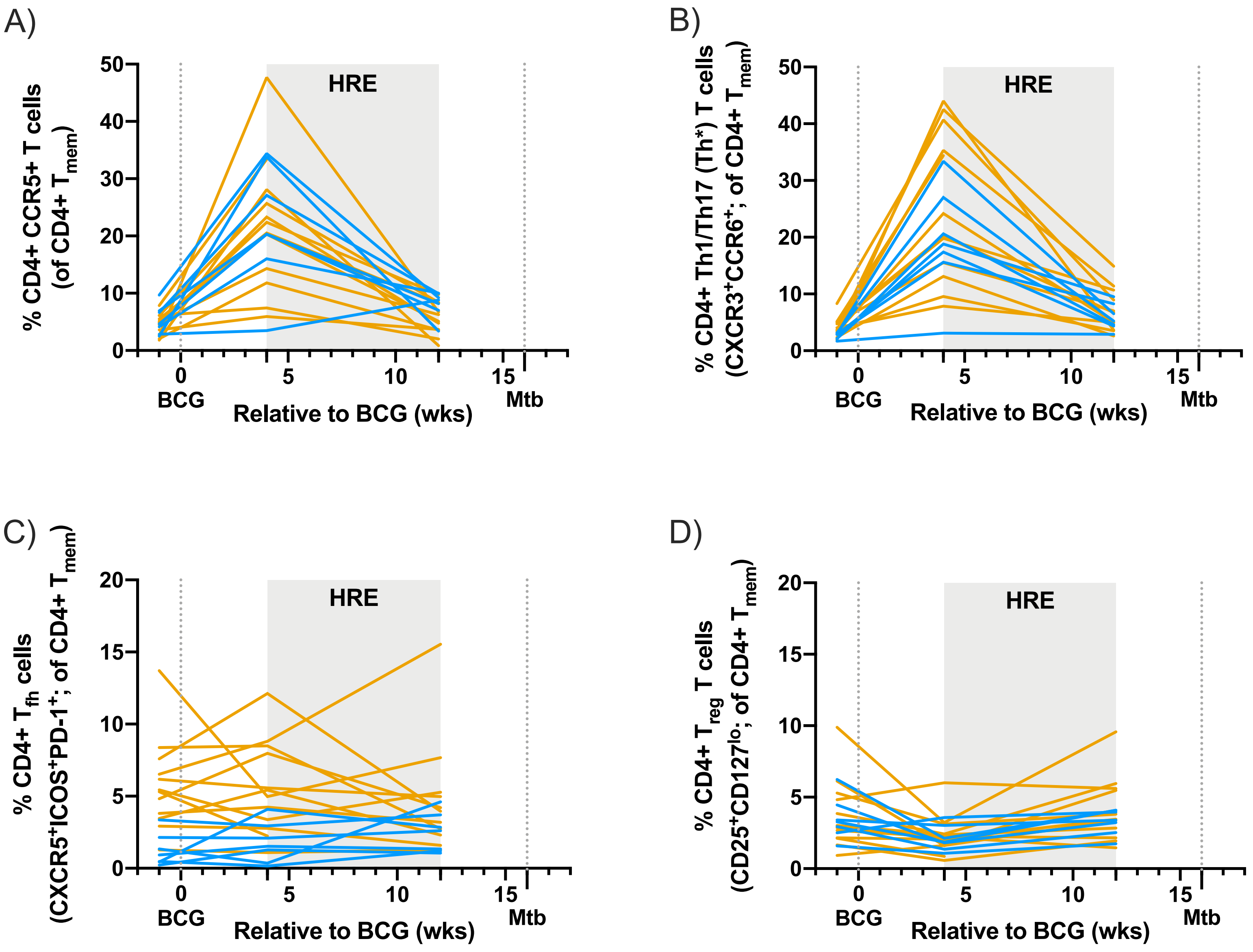

Supplementary Data 8. CD4+ T cell phenotype in PBMC after vaccination in individual animals. A) Frequency of CD4+ CCR5+ T cells in PBMC relative to BCG vaccination. B) Frequency of CD4+ Th1/Th17 (Th*) T cells ($CXCR3^+CCR6^+$) in PBMC relative to BCG vaccination. C) Frequency of CD4+ T_{fh} T cells ($CXCR5^+ICOS^+PD\text{-}1^+$) in PBMC relative to BCG vaccination. D) Frequency of CD4+ T_{reg} T cells ($CD25^+CD127^{lo}$) in PBMC relative to BCG vaccination. A-D) Lines indicate individual animals of SIV-naïve (light blue) and SIV+ (gold) vaccinated groups. SIV-naïve vaccinated animals (IV BCG) : pre BCG (n = 7), 4 wks post BCG (n = 7), and 12 wks post BCG (n = 7). SIV+ vaccinated animals (SIV/IV BCG): pre BCG (n = 12), 4 wks post BCG (n = 12), and 12 wks post BCG (n = 11).
