# Supplementary material for: Intravenous Bacille Calmette–Guérin vaccination protects simian immunodeficiency virus-infected macaques from tuberculosis
Source: Nat Microbiol. 2023 Oct 9;8(11):2080–92. doi: 10.1038/s41564-023-01503-x (PMC10627825; doi:10.1038/s41564-023-01503-x)
Supplement: Supplementary file 2 — Reporting summary [file 41564_2023_1503_MOESM2_ESM.pdf]

nature portfolio

Corresponding author(s): Drs. Erica Larson and Charles Scanga

Last updated by author(s): Sep 6, 2023

Reporting Summary

Nature Portfolio wishes to improve the reproducibility of the work that we publish. This form provides structure for consistency and transparency in reporting. For further information on Nature Portfolio policies, see our Editorial Policies and the Editorial Policy Checklist.

Statistics

For all statistical analyses, confirm that the following items are present in the figure legend, table legend, main text, or Methods section.

n/a	Confirmed	
☐	☒	The exact sample size (*n*) for each experimental group/condition, given as a discrete number and unit of measurement
☐	☒	A statement on whether measurements were taken from distinct samples or whether the same sample was measured repeatedly
☐	☒	The statistical test(s) used AND whether they are one- or two-sided<br>*Only common tests should be described solely by name; describe more complex techniques in the Methods section.*
☒	☐	A description of all covariates tested
☐	☒	A description of any assumptions or corrections, such as tests of normality and adjustment for multiple comparisons
☐	☒	A full description of the statistical parameters including central tendency (e.g. means) or other basic estimates (e.g. regression coefficient) AND variation (e.g. standard deviation) or associated estimates of uncertainty (e.g. confidence intervals)
☐	☒	For null hypothesis testing, the test statistic (e.g. *F*, *t*, *r*) with confidence intervals, effect sizes, degrees of freedom and *P* value noted<br>*Give P values as exact values whenever suitable.*
☒	☐	For Bayesian analysis, information on the choice of priors and Markov chain Monte Carlo settings
☒	☐	For hierarchical and complex designs, identification of the appropriate level for tests and full reporting of outcomes
☒	☐	Estimates of effect sizes (e.g. Cohen's *d*, Pearson's *r*), indicating how they were calculated

Our web collection on statistics for biologists contains articles on many of the points above.

Software and code

Policy information about availability of computer code

Data collection	SpectroFlo v2.2; FACS Diva v9.3; ImmunoSpot software v5.1; Promega GloMax Multi detection system software v1.3.2; Roche LightCycler 480 software v1.5; OsiriX MD software v12.5.2.
Data analysis	FlowJo v10.1 & v10.8.1; Prism v8.2.1

For manuscripts utilizing custom algorithms or software that are central to the research but not yet described in published literature, software must be made available to editors and reviewers. We strongly encourage code deposition in a community repository (e.g. GitHub). See the Nature Portfolio guidelines for submitting code & software for further information.

Data

Policy information about availability of data

All manuscripts must include a data availability statement. This statement should provide the following information, where applicable:

- Accession codes, unique identifiers, or web links for publicly available datasets
- A description of any restrictions on data availability
- For clinical datasets or third party data, please ensure that the statement adheres to our policy

All relevant data are available from the corresponding author upon reasonable request.

Research involving human participants, their data, or biological material

Policy information about studies with human participants or human data. See also policy information about sex, gender (identity/presentation), and sexual orientation and race, ethnicity and racism.

Reporting on sex and gender	N/A
Reporting on race, ethnicity, or other socially relevant groupings	N/A
Population characteristics	N/A
Recruitment	N/A
Ethics oversight	N/A

Note that full information on the approval of the study protocol must also be provided in the manuscript.

Field-specific reporting

Please select the one below that is the best fit for your research. If you are not sure, read the appropriate sections before making your selection.

☒ Life sciences ☐ Behavioural & social sciences ☐ Ecological, evolutionary & environmental sciences

For a reference copy of the document with all sections, see nature.com/documents/nr-reporting-summary-flat.pdf

Life sciences study design

All studies must disclose on these points even when the disclosure is negative.

Sample size	We used total thoracic bacterial burden (log10) as the primary outcome variable with a pooled standard deviation of 1.09 (calculated by averaging standard deviations of all groups) for a two-sided test and adjusted the type I error for two comparisons (alpha = 0.025). For the unvaccinated (n = 8) and IV BCG (n = 7) comparison, we obtained 83.0% power to detect a mean difference of 2 in bacterial burden. For the SIV/Unvaccinated (n = 11) and SIV/IV BCG (n = 12) comparison, we obtained 97.0% power to detect a mean difference of 2.
Data exclusions	Two IV BCG-vaccinated animals (82-18 & 192-18) did not reach Mtb challenge due to reasons unrelated to BCG. For this reason, we were unable to assess vaccine efficacy and related immune parameters and these animals were excluded from all analyses.<br><br>For BAL analyses, some time points either did not have enough cells for all tests in which they were prioritized for ICS, had low cell viability (< 70%), or displayed autofluorescence that reduced the signal to noise ratio rendering gating positive populations difficult. For these reasons, these data were excluded from BAL analyses.
Replication	Due to limitations of NHP studies (i.e., cost and animal welfare), we conducted a power analysis to detect a meaningful effect size.
Randomization	Animals were arbitrarily assigned to groups.
Blinding	Technicians that performed qPCR for plasma viral loads were blinded from treatment group. Prosectors who conducted the necropsies, image analysts for PET/CT, the pathologist and technicians who processed samples were blinded from treatment group as well. Flow cytometry and Antibody ELISA analyses was performed by investigators (E.C.L., A.L.E.C., & A.J.B.) who were intimately familiar with all aspects of the study including group assignments which made it impossible to blind.

Reporting for specific materials, systems and methods

We require information from authors about some types of materials, experimental systems and methods used in many studies. Here, indicate whether each material, system or method listed is relevant to your study. If you are not sure if a list item applies to your research, read the appropriate section before selecting a response.

Materials & experimental systems

n/a	Involved in the study
☐	☒ Antibodies
☒	☐ Eukaryotic cell lines
☒	☐ Palaeontology and archaeology
☐	☒ Animals and other organisms
☒	☐ Clinical data
☒	☐ Dual use research of concern
☒	☐ Plants

Methods

n/a	Involved in the study
☒	☐ ChIP-seq
☐	☒ Flow cytometry
☒	☐ MRI-based neuroimaging

Antibodies

Antibodies used

Flow cytometry:
PBMC ICS
CD107a BUV395 (BD; Cat No. 565113; Clone eBioH4A3; dilution: 1:25) and CD154 BV421 (BD; Cat No. 563886; Clone TRAP1; dilution: 1:25) was added during the BFA and monensin step 2 hours after the stimulators were added. After the 14-hour stimulation, cells were stained for viability using LIVE/DEAD™ Fixable Blue Dead Cell Stain Kit (Invitrogen; Cat No. L23105; dilution: 1:1000). For the surface antibody cocktail, the following antibodies were used: Vg9 FITC (Invitrogen; Cat No. TCR2720; Clone 7A5; dilution: 1:40); CCR4 BV510 (Biolegend; Cat No. 359416; Clone L291H4; dilution: 1:50); CXCR3 BV605 (Biolegend; Cat No. 353728; Clone G025H7; dilution: 1:20); PD-1 BV711 (Biolegend; Cat No. 329928; Clone EH12.2H7; dilution: 1:20); CD4 BUV496 (BD; Cat No. 750591; Clone L200; dilution: 1:400); CCR6 BUV737 (BD; Cat No. 612780; Clone 11A9; dilution: 1:25); CD8 BUV805 (BD; Cat No. 564912; Clone SK1; dilution: 1:40); CCR7 Alexa Fluor 700 (BD; Cat No. 561143; Clone 150503; dilution: 1:20); CD3 APC-Cy7 (BD; Cat No. 557757; Clone SP34-2; dilution: 1:200); CD28 PE-Cy5 (BD; Cat No. 555730; Clone CD28.2; dilution: 1:20); and CD45RA PE-Cy7 (BD; Cat No. 561216; Clone 5H9; dilution: 1:160). For the intracellular antibody cocktail, the following antibodies were used: IL-17A BV570 (Biolegend; Cat No. 512324; Clone BL168; dilution: 1:25); TNF BV650 (BD; Cat No. 563418; Clone MAb11; dilution: 1:20); IL-2 BV750 (BD; Cat No. 566361; Clone MQ1-17H12; dilution: 1:50); IFNg APC (BD; Cat No. 554702; Clone B27; dilution: 1:200); CD69 ECD (Beckman; Cat No. 6607110; Clone TP1.55.3; dilution: 1:100); Granulysin PE (Biolegend; Cat No. 348004; Clone DH2; dilution: 1:20); and Granzyme B PE-Cy5.5 (Invitrogen; Cat No. GRB18; Clone GB11; dilution: 1:160). All samples were stained in a final volume of 50 uL.

PBMC Phenotype
Cells were with MR-1 tetramer BV421 loaded with 5-OPRU (NIH Tetramer Core; dilution: 1:50) for 30 minutes at 4C. Viability was subsequently stained using LIVE/DEAD™ Fixable Blue Dead Cell Stain Kit (Invitrogen; Cat No. L23105; dilution: 1:1000). For the surface antibody cocktail, the following antibodies were used: Vg9 FITC (Invitrogen; Cat No. TCR2720; Clone 7A5; dilution: 1:40); PD-1 BB660 (BD; custom order; clone EH12.1; dilution: 1:50); CCR7 BB700 (BD; Cat No. 566437; Clone 3D12; dilution 1:20); CD127 BV510 (Biolegend; Cat No. 351332; Clone A019D5; dilution: 1:40); CD20 BV570 (Biolegend; Cat No. 302331; Clone 2H7; dilution: 1:40); CXCR5 Super Bright 600 (Invitrogen; Cat No. 63-9185-42; Clone MU5UBEE; dilution: 1:10); CD16 BV650 (BD; Cat No. 563692; Clone 3G8; dilution: 1:80); CXCR3 BV711 (Biolegend; Cat No. 353732; Clone G025H7; dilution 1:10); CD14 BV785 (Biolegend; Cat No. 301839; Clone M5E2; dilution 1:80); CD45RA BUV395 (BD; Cat No. 740315; Clone 5H9; dilution: 1:320); CD4 BUV496 (BD; Cat No. 750591; Clone L200; dilution: 1:400); CD25 BUV563 (BD; Cat No. 612918; Clone 2A3; dilution: 1:40); CCR6 BUV737 (BD; Cat No. 612780; Clone 11A9; dilution: 1:25); CD8 BUV805 (BD; Cat No. 564912; Clone SK1; dilution: 1:40); CCR5 APC (BD; Cat No. 564912; Clone 3A9; dilution: 1:10); ICOS R718 (BD; Cat No. 566990; Clone C398.4A; dilution: 1:160); CD3 APC-Cy7 (BD; Cat No. 557757; Clone SP34-2; dilution: 1:50); gd TCR PE (ThermoFisher; Cat No. MHGD04; Clone 5A6.E9; dilution: 1:10); NKG2a ECD (Beckman; Cat No. IM99512; Clone Z199; dilution: 1:40); CD123 PE-Cy5 (Biolegend; Cat No. 306008; Clone 6H6; dilution: 1:160); HLA-DR PE-Cy5.5 (Life Tech; Cat No. MHLDR18; Clone TU36; dilution: 1:400); and CD11c PE-Cy7 (Biolegend; Cat No. 301608; Clone 3.9; dilution: 1:100). All samples were stained in a final volume of 50 uL.

BAL ICS
CD107a PerCP-eFluor710 (eBioscience; Cat No. 46-1079-42; Clone eBioH4A3; dilution: 1:100) was added during the BFA and monensin step 2 hours after the stimulators were added. After the 14-hour stimulation, cells were washed and stained with MR-1 tetramer BV421 loaded with 5-OPRU (NIH Tetramer Core; dilution: 1:50) for 30 minutes at 4C. Viability was subsequently stained using Zombie NIR™ Fixable Viability Kit (Biolegend; Cat No. 423106; dilution 1:200). For the surface antibody cocktail, the following antibodies were used: CXCR5 Super Bright 436 (eBioscience; Cat No. 62-9185-42; Clone MU5UBEE; dilution: 1:33); CD8 Pacific Blue (Dako; Cat No. PB984; Clone DK25; dilution: 1:33); CCR7 BV480 (BD; Cat No. 566099; Clone 3D12; dilution: 1:40); CD45 BV510 (BD; Cat No. 563530; Clone DO58-1283; dilution: 1:66); PD-1 BV605 (Biolegend; Cat No. 329924; Clone EH12.2H7; dilution 1:33); CXCR3 BV711 (Biolegend; Cat No. 353732; Clone G025H7; dilution 1:33); CD127 BV785 (Biolegend; Cat No. 351330; Clone A019D5; dilution: 1:20); CD25 BB515 (BD; Cat No. 564467; Clone 2A3; dilution 1:33); Vg9 FITC (Invitrogen; Cat No. TCR2720; Clone 7A5; dilution 1:66); CD28 PE-Cy5 (BD; Cat No. 555730; Clone CD28.2; dilution 1:20); CCR6 PerCP-Cy5.5 (BD; Cat No. 560467; Clone 11A9; dilution 1:25); CD45RA PE-Cy7 (BD; Cat No. 561216; Clone 5H9; dilution 1:25); ICOS Alexa Fluor 647 (Biolegend; Cat No. 313516; Clone C398.4A; dilution 1:33); CD103 conjugated to Alexa Fluor 680 (Beckman Coulter; Cat No. IM0318; Clone 2G5; dilution 1:166); and CD4 Alexa Fluor 700 (eBioscience; Cat No. 56-0048-41; Clone OKT4; dilution 1:200). For the intracellular antibody cocktail, the following antibodies were used: IL-17A BV570 (Biolegend; Cat No. 512324; Clone BL168; dilution 1:25); TNF BV650 (Biolegend; Cat No. 502937; Clone MAb11; dilution 1:40); IL-2 PE (Biolegend; Cat No. 500307; Clone MQ1-17H12; dilution 1:33); CD69 PE-Dazzle594 (Biolegend; Cat No. 310942; Clone FN50; dilution 1:25); IFNg APC (BD; Cat No. 562017; Clone B27; dilution 1:66); and CD3 APC-Cy7 (BD; Cat No. 557757; Clone SP34-2; dilution 1:25). All samples were stained in a final volume of 100 uL for surface and intracellular antibody cocktails.

BAL Phenotype
Cells were with MR-1 tetramer BV421 loaded with 5-OPRU (NIH Tetramer Core; dilution: 1:50) for 30 minutes at 4C. Viability was subsequently stained using Zombie NIR ™ Fixable Viability Kit (Biolegend; Cat No. 423106; dilution 1:200). For the surface antibody cocktail, the following antibodies were used: CD16 Super Bright 436 (eBioscience; Cat No. 62-0166-41; Clone 3G8; dilution 1:33); CD8

Pacific Blue (Dako; Cat No. PB984; Clone DK25; dilution: 1:33); CCR7 BV480 (BD; Cat No. 566099; Clone 3D12; dilution: 1:40); CD45 BV510 (BD; Cat No. 563530; Clone DO58-1283; dilution: 1:66); CD20 BV570 (Biolegend; Cat No. 302331; Clone 2H7; dilution 1:66); PD-1 BV605 (Biolegend; Cat No. 329924; Clone EH12.2H7; dilution 1:33); CXCR3 BV711 (Biolegend; Cat No. 353732; Clone G025H7; dilution 1:33); CD14 BV785 (Biolegend; Cat No. 301839; Clone M5E2; dilution 1:25); CCR5 BB515 (BD; Cat No. 564512; Clone 3A9; dilution 1:33); Vg9 FITC (Invitrogen; Cat No. TCR2720; Clone 7A5; dilution 1:66); CD66abce PE (Miltenyi; Cat No. 130-117-811; Clone TET2; dilution 1:66); CD69 PE-Dazzle594 (Biolegend; Cat No. 310942; Clone FN50; dilution 1:25); CD11b PE-Cy5 (Biolegend; Cat No. 301307; Clone ICRF44; dilution 1:25); CD11c PE-Cy7 (Biolegend; Cat No. 301607; Clone 3.9; dilution: 1:100); NKG2a APC (Beckman; Cat No. A60797; Clone Z199; dilution 1:66); CCR4 Alexa Fluor 647 (BD; Cat No. 557863; Clone 1G1; dilution 1:50); CD4 Alexa Fluor 700 (eBioscience; Cat No. 56-0048-41; Clone OKT4; dilution 1:200); and CD163 APC-Cy7 (Biolegend; Cat No. 333622; Clone GH1-61; dilution 1:66). All samples were stained in a final volume of 100 uL.

Antibody ELISAs
The following antibodies and their final concentrations were used for ELISAs: goat anti-Monkey IgG heavy and light chain antibody – HRP conjugated (Bethyl Laboratories; Cat No. A140-102P; 50 ng/mL); goat anti-Monkey IgA (a chain) antibody – HRP conjugated (Rockland Immunochemicals Inc.; Cat No. 617-103-006; 0.1 ug/mL); and goat anti-Monkey IgM (a chain) antibody – HRP conjugated (Sera Care; Cat No. 5220-0334; 0.4 ug/mL).

All reagents are commercially available with the exception of goat anti-Monkey IgA and goat anti-Monkey IgM, which have since been discontinued. We are using alternative antibodies for current studies to detect IgA and IgM antibodies, which are comparable. Vendor information for these reagents are available upon request.

Validation

All antibodies for flow cytometry are extensively validated by the vendors and further validated by the investigators of this study.
BD website: https://www.bdbiosciences.com/en-us/products/reagents/flow-cytometry-reagents/research-reagents
Biolegend website: https://www.biolegend.com/en-us/quality
Invitrogen website: https://www.thermofisher.com/us/en/home/life-science/cell-analysis/flow-cytometry/antibodies-for-flow-cytometry.html
Beckman website: https://www.beckman.com/reagents/coulter-flow-cytometry
Miltenyi website:https://www.miltenyibiotec.com/US-en/products/macs-antibodies/antibodies-for-flow-cytometry.html

For panel design and optimization, single color controls were initially used to obtain the optimal signal to noise. Due to the overlap between humans and NHPs, clone compatibility was referenced according to the reactivity database on the NHP Reagent Resource website (https://www.nhpreagents.org/ReactivityDatabase), a reagent database that is composed of over 20 years of research contributed data. Antibody titration was performed using NHP PBMC starting with the vendor's recommended concentration and then titrating down the amount of antibody to the concentrations listed. Fluorescence minus one experiments were used to further optimize signal to noise and minimize interactions between antibody-linked fluorochromes.

Antibodies for used for ELISAs were validated previously in Darrah et al. 2020 and further optimized in the O'Connor Lab.

Animals and other research organisms

Policy information about studies involving animals; ARRIVE guidelines recommended for reporting animal research, and Sex and Gender in Research

Laboratory animals

Adult Mauritian cynomolgus macaques (Macaca fascicularis; age > 4 years old) were purchased from Bioculture US (Immokalee, FL).

Wild animals

No wild animals were used in the study.

Reporting on sex

A total of 40 animals were used for this study. Of those, 33 were contemporaneous and 7 were historical controls. We used both male and female animals, although their distribution was unequal. Of the contemporaneous animals, only 6 were female; of the historical controls, only 2 were female. The reason for this sex imbalance was supply and demand- Bioculture simply had more male animals available. Nonetheless, we have no reason to suspect that the findings reported here apply only to males. Indeed, there was no discernable effect of sex on our findings. Importantly, Extended Data Table 1 lists each animal and, among other information, indicates whether each was male or female.

Field-collected samples

No field collected samples were used in the study.

Ethics oversight

Animal protocols and procedures were approved by the University of Pittsburgh Institutional Animal Care and Use Committee (IACUC) which adheres to guidelines established in the Animal Welfare Act and the Guide for the Care and Use of Laboratory Animals, as well as the Weatherall Report (8th Edition). The University is fully accredited by AAALAC (accreditation number 000496), and its OLAW animal welfare assurance number is D16-00118. The IACUC reviewed and approved the study protocols 15035407 and 18032418, under Assurance Number A3187-01 and D16-00118, respectively.

Note that full information on the approval of the study protocol must also be provided in the manuscript.

Flow Cytometry

Plots

Confirm that:

☒ The axis labels state the marker and fluorochrome used (e.g. CD4-FITC).

☒ The axis scales are clearly visible. Include numbers along axes only for bottom left plot of group (a 'group' is an analysis of identical markers).

☒ All plots are contour plots with outliers or pseudocolor plots.

☒ A numerical value for number of cells or percentage (with statistics) is provided.

Methodology

Sample preparation	PBMC were isolated from blood using Ficoll-Paque PLUS gradient separation (GE Healthcare Biosciences. Single-cell suspensions were cryopreserved in FBS containing 10% DMSO in liquid nitrogen. BAL wash fluid (4 x 10 mL washes of PBS) was pelleted and an aliquot of wash fluid (15 mL) was cryopreserved. The remaining cells were resuspended into ELISpot media (RPMI 1640, 10% heat-inactivated human albumin, 1% L-glutamine, and 1% HEPES) and counted. BAL cells were then divided into their appropriate flow cytometry assay depending on cell yield.<br><br>Longitudinal BAL and PBMC samples were stained for leukocyte composition (phenotype) or antigen-specific T cell responses. In general, BAL cells were stained immediately and PBMC were cryopreserved and batch-analyzed at the end of the study. For BAL analyses, cells were counted and aliquoted into a 96-well plate for either phenotype or intracellular cytokine responses (1 x 10^6 cells/well). In cases where total BAL cell counts were low, cells were prioritized for the 14-hour stimulation assay. For the phenotype panel, cells were reconstituted in 500 nM dasatinib in ELISpot media to improve MR-1 (5-OP-RU; BV421) tetramer staining. Cells were incubated with MR1 tetramer (NIH Tetramer Core Facility, Atlanta, GA) for 30 minutes at 4C. The MR1 technology was developed jointly by Dr. James McCluskey, Dr. Jaime Rossjohn, and Dr. David Fairlie, and the material was produced by the NIH Tetramer Core Facility as permitted to be distributed by the University of Melbourne. Cells were then washed and stained for viability (Zombie Live/Dead near IR; Invitrogen) for 10 minutes at room temperature. Cells were then washed, incubated with surface antibody cocktail for 20 minutes at 4C, and fixed with 1% paraformaldehyde for 15 minutes. For the intracellular cytokine assay, cells were aliquoted (1 x 10^6 cells/well) into either a media control well, purified protein derivative well (PPD; 20 ug/mL; AJ Vaccines), or ESAT-6/CFP-10 overlapping peptide pools (1 ug/mL each; BEI Resources). Stimulators were added and incubated for 2 hours, then brefeldin A (1 ug/mL; Biolegend; Cat No. 420601) and monensin (1 ug/mL; Biolegend; Cat No. 420701) was added for the remainder of the stimulation time (12 hours). Cells were washed and surface markers were stained similarly as described for the phenotype panel. However, after PFA fixation, cells were permeabilized with BD Cytofix/Cytoperm™ (BD; Cat No. 554714) for 10 minutes at room temperature. Intracellular markers were stained for 20 minutes at room temperature. Stained BAL cells were run within two days of staining. Flow cytometry of BAL was performed using a Cytek Aurora (BD; SpectroFlo v2.2). FCS files were analyzed using FlowJo software for Macintosh (version 10.1).<br><br>For PBMC analyses, cells were divided to be stained for phenotype or 14-hour stimulation. PBMC stimulators include: H37Rv whole cell lysate (20 ug/mL, BEI Resources) and ESAT-6/CFP-10 peptide pools (1 ug/mL each). Cell were stained as follows: cells were stained with MR-1 tetramer for 20 minutes; washed twice with PBS/BSA (0.1%); then stained with viability dye for 20 minutes; washed twice with PBS/BSA (0.1%) and incubated with human FcR blocking reagent (Miltenyi); surface markers were stained for 20 minutes and washed three times with PBS/BSA (0.1%); cells were permeabilized with BD Cytofix/ Cytoperm™ (BD; Cat No. 554714) for 20 minutes and intracellular markers were stained for 30 minutes. Data was acquired on a modified BD LSR Fortessa (FACS Diva v9.3) and analyzed in FlowJo software for Macintosh (version 10.8.1).
Instrument	Cytek Aurora and BD LSR Fortessa
Software	SpectroFlo (Cytek, v2.2), FACS Diva (BD, version 9.3), FlowJo (v10.1 & v10.8.1)
Cell population abundance	We stained 1x10^6 cells for all assays, when possible. Cell population abundance is reflective of the populations present after sample collection, as these samples were not sorted for the assays described. Cell population abundance is indicated as frequencies in source data 2, 3, and 4.
Gating strategy	See Supplemental Data 5-8 for gating strategies.<br><br>For populations identified by the BAL and PBMC phenotype panels, most positive populations were clearly separated from the negative populations (> 2 decades). For MR1 5-OP-RU staining, a control tetramer (6-FP) was used to determine gate placement for the positive population. For the ICS panels, gating for cytokines were set based on unstimulated samples (media only). For markers that did not have a clearly separated population, fluorescence minus one samples (FMOs) were used to gain a general sense of the location of the positive population. Gates were drawn along the outer edge of the negative population.

☒ Tick this box to confirm that a figure exemplifying the gating strategy is provided in the Supplementary Information.
